# Wild-type Cu/Zn-superoxide dismutase is misfolded in cerebrospinal fluid of sporadic amyotrophic lateral sclerosis

**DOI:** 10.1186/s13024-019-0341-5

**Published:** 2019-11-19

**Authors:** Eiichi Tokuda, Yo-ichi Takei, Shinji Ohara, Noriko Fujiwara, Isao Hozumi, Yoshiaki Furukawa

**Affiliations:** 1 Laboratory for Mechanistic Chemistry of Biomolecules, Department of Chemistry, Keio University, Yokohama, 223-8522 Japan; 2 Department of Neurology, Matsumoto Medical Center, Matsumoto, 399-0021 Japan; 3 Department of Neurology, Iida Hospital, Iida, 395-8505 Japan; 4 Department of Biochemistry, Hyogo College of Medicine, Nishinomiya, 663-8501 Japan; 5 Laboratory of Medical Therapeutics and Molecular Therapeutics, Gifu Pharmaceutical University, Gifu, 501-1196 Japan; 6 Department of Neurology and Geriatrics, Gifu University Graduate School of Medicine, Gifu, 501-1194 Japan

**Keywords:** Amyotrophic lateral sclerosis (ALS), Cerebrospinal fluid (CSF), Cu/Zn-superoxide dismutase (SOD1), Protein misfolding

## Abstract

**Background:**

A subset of familial forms of amyotrophic lateral sclerosis (ALS) are caused by mutations in the gene coding Cu/Zn-superoxide dismutase (SOD1). Mutant SOD1 proteins are susceptible to misfolding and abnormally accumulated in spinal cord, which is most severely affected in ALS. It, however, remains quite controversial whether misfolding of wild-type SOD1 is involved in more prevalent sporadic ALS (sALS) cases without *SOD1* mutations.

**Methods:**

Cerebrospinal fluid (CSF) from patients including sALS as well as several other neurodegenerative diseases and non-neurodegenerative diseases was examined with an immunoprecipitation assay and a sandwich ELISA using antibodies specifically recognizing misfolded SOD1.

**Results:**

We found that wild-type SOD1 was misfolded in CSF from all sALS cases examined in this study. The misfolded SOD1 was also detected in CSF from a subset of Parkinson’s disease and progressive supranuclear palsy, albeit with smaller amounts than those in sALS. Furthermore, the CSF samples containing the misfolded SOD1 exhibited significant toxicity toward motor neuron-like NSC-34 cells, which was ameliorated by removal of the misfolded wild-type SOD1 with immunoprecipitation.

**Conclusions:**

Taken together, we propose that misfolding of wild-type SOD1 in CSF is a common pathological process of ALS cases regardless of *SOD1* mutations.

## Background

Amyotrophic lateral sclerosis (ALS) causes adult-onset, progressive degeneration of motor neurons, leading to muscle weakness, paralysis, and death usually within 3–5 years of diagnosis [1]. No effective cures for ALS are currently available. While the majority (*approx*. 90%) of total ALS cases are sporadic, a family history has been confirmed in the remaining cases [2]. Increasing numbers of genes responsible for ALS have been identified [1]; among those, mutations in the gene coding Cu/Zn-superoxide dismutase (SOD1) account for approximately 20% of familial cases (*SOD1*-ALS) [3] and a small percentage of sporadic cases [4, 5]. Several lines of evidence have supported a toxic gain-of-function mechanism where mutation-induced misfolding of SOD1 associates with toxicity causing degeneration of motor neurons [6].

Even in the absence of any mutations, wild-type SOD1 can be misfolded into abnormal oligomers and insoluble aggregates upon demetallation, disulfide reduction, and/or oxidative modification in vitro [7–9]. Some researchers have hence expected misfolding of wild-type SOD1 as a pathological change in sporadic ALS (sALS) without *SOD1* mutations. Indeed, immunoreactivities of misfolded SOD1-specific antibodies were observed in spinal motor neurons of ALS patients without *SOD1* mutations [10–13], and overexpression of wild-type SOD1 in mice caused ALS-like symptoms [14]. Abnormal changes of wild-type SOD1 have been reported also in the other neurodegenerative diseases such as Alzheimer’s disease (AD) and Parkinson’s disease (PD) [15, 16]. Nonetheless, several studies have not supported the immunostaining of motor neurons of sALS with misfolded SOD1-specific antibodies [17–19]. Depending upon experimental protocols such as antigen retrieval, immunoreactivity with misfolded SOD1-specific antibodies could be false positive in motor neurons of sALS [13, 20]. It hence remains quite controversial whether wild-type SOD1 is involved in the pathogenesis of sALS.

In contrast to the ambiguous characterization of misfolded SOD1 in sALS, several studies have pointed to toxicity of wild-type SOD1 toward cultured motor neurons in pathological conditions. For example, SOD1 immunopurified from spinal cord of sALS cases but not of a control was protease-resistant [12] and found to inhibit the anterograde axonal transport in a manner resembling that of mutant SOD1 [10]. Also, astrocytes generated from sALS patients were toxic to motor neurons, and this toxicity was significantly reduced by shRNA-based suppression of wild-type SOD1 expression in the sALS astrocytes [21]. Given that culture media of the astrocytes from sALS patients killed motor neurons [21], wild-type SOD1 might be involved in the extracellular release of as-yet-unidentified toxic factors and thereby contribute to the pathogenesis of sALS.

Notably, SOD1 itself is secreted from a range of cell types [22], and abnormal forms of SOD1 in vitro can exert their toxicity to cultured cells [23, 24]. SOD1 species secreted from neurons and glia are also expected to move into interstitial fluid and then spread over the central nervous system via cerebrospinal fluid (CSF); indeed, SOD1 is a constituent of CSF. While there appeared to be no difference in amounts of SOD1 in CSF between ALS and non-ALS cases [25–27], CSF from sALS patients have been reported to induce degeneration of a motor neuronal cell line [28]. Furthermore, it was recently reported that wild-type SOD1 in CSF was oxidized at its Cys residue (sulfenylation at Cys111) in some sALS cases [29]. We hence expected that even in the absence of pathogenic mutations, wild-type SOD1 in CSF is conformationally affected under pathological conditions of sALS.

In this study, we utilized a panel of antibodies that can specifically recognize non-native conformations of SOD1 and found misfolded forms of SOD1 in CSF from all ALS cases examined including twenty sALS cases and one *SOD1*-ALS case. CSF from a subset of PD and progressive supranuclear palsy (PSP) cases was also found to contain the misfolded SOD1, albeit with smaller amounts. Furthermore, we confirmed the toxicity of the CSF samples containing the misfolded SOD1 toward motor neuron-like cells, NSC-34, and quite notably, the toxicity was significantly ameliorated by absorbing the misfolded SOD1 with a misfolded SOD1-specific antibody C4F6. We thus propose that misfolding of wild-type SOD1 in CSF is the pathological change commonly occurring in ALS cases regardless of *SOD1* mutations.

## Methods

### Human cases

Human cases examined in this study were twenty sALS cases, one familial *SOD1*-ALS case, and forty non-ALS cases. Detailed information on these cases are summarized in Tables 1 and 2. All tissues and CSF samples examined in this study were obtained with written informed consent at either Matsumoto Medical Center (C-1 – C-33 and ALS1 – ALS13 in Tables 1 and 2) or Gifu University Hospital (C-34 – C-40 and ALS14 – ALS21 in Tables 1 and 2) in Japan. Collection of tissue/CSF samples and their use for the research were approved by the institutional review board for research ethics of Matsumoto Medical Center (30–2), Gifu Pharmaceutical University (29–48), Gifu University (27–120), and Keio University (30–110), Japan. All procedures performed in our studies involving human participants were in accordance with the ethical standards of the Matsumoto Medical Center, Gifu Pharmaceutical University, and Keio University research committees and also with the 1964 Helsinki declaration and its later amendments or comparable ethical standards. Most of the CSF samples examined here were collected for diagnostic purpose by lumbar puncture from patients; therefore, the age at collection in Tables 1 and 2 can be regarded as the age at disease onset. CSF from ALS and non-ALS cases was collected with a same general procedure and then stored at -70 °C until use. To avoid repeated freeze/thaw cycles of the CSF samples, the samples were aliquoted in small volumes. While large increases in pH have been pointed out in weakly buffered CSF during storage [30], the pH of our CSF samples ranged from 7 to 8. Also, our CSF samples were diluted in either Tris or phosphate-based buffer at pH 7.0 just prior to experiments (see below). Total protein concentrations of the CSF samples were determined using Micro BCA™ Assay Kit (Thermo Scientific) and shown in Tables 1 and 2. There was no statistical difference in the total protein concentrations in CSF between ALS and non-ALS (*P* = 0.10, Student’s *t*-test, Additional file 2: Figure S1), while the medians were different (735 μg/mL and 611 μg/mL in non-ALS and ALS, respectively).

**Table 1:**
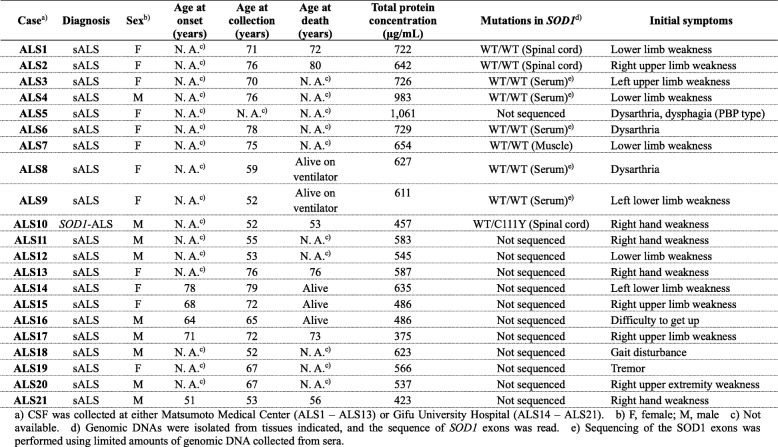
Information on CSF samples of human non-ALS cases in this study

**Table 2:**
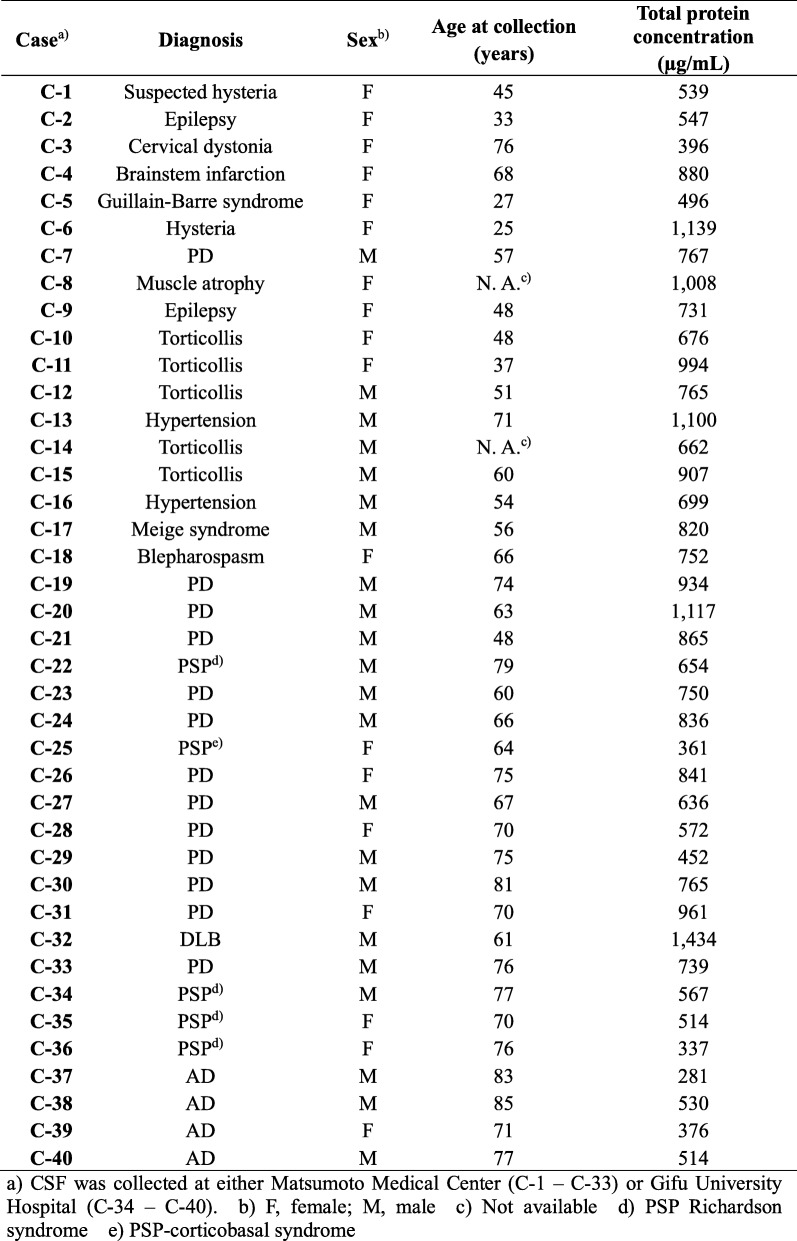
Information on CSF samples of human ALS cases in this study

### DNA sequencing

Genomic DNA was isolated from the samples (spinal cord, sera, and skeletal muscle) using GenoPlus Genomic DNA Mini (Viogene) according to the manufacturer’s protocol. Each of five exons in the *SOD1* gene was amplified with PCR using KOD FX Neo DNA polymerase (TOYOBO). Primers used for amplification of the exons are summarized in Additional file 1: Table S1. For amplification of the exon 2 fragment, a stepdown PCR was performed: a pre-denature step at 98 °C for 2 min, five cycles of denature (98 °C, 10 s) and extension (74 °C, 60 s), five cycles of denature (98 °C, 10 s) and extension (72 °C, 60 s), five cycles of denature (98 °C, 10 s) and extension (70 °C, 60 s), and twenty cycles of denature (98 °C, 10 s) and extension (68 °C, 60 s). For the other exon fragments, a 3-step PCR was performed, which was comprised of a pre-denature step at 94 °C for 2 min followed by 35 cycles of denature (98 °C, 10 s), annealing (62 °C, 30 s), and extension (68 °C, 2 min). The amplified fragments containing the exons were purified by an ethanol precipitation method, treated with ExoSAP-IT (Thermo Fisher Scientific) to remove the primers for PCR, and then further purified with Gel/PCR Extraction Kit (FastGene). DNA sequencing of those purified fragments was performed using a primer for sequencing (Additional file 1: Table S1, Eurofins Genomics).

An abnormal expansion of a noncoding GGGGCC repeat within *C9ORF72* gene, which has been identified as a major cause of ALS in Caucasian patients [31], was also analyzed by a PCR using the primers flanking the repeat region (Additional file 1: Table S1, Eurofins Genomics) [32]. The PCR was performed by using Advantage® GC Genomic LA Polymerase Mix with the manufacturer’s instructions, and an agarose gel electrophoretic analysis of the amplified fragments showed no smears in a high molecular weight region (data not shown), confirming no pathogenic repeat expansion in *C9ORF72* gene of our ALS cases examined here. All patients in this study were Japanese, and this supports a previous report showing that ALS cases with abnormal repeat expansion in *C9ORF72* gene are quite rare in Asian patients including Japanese [33].

### Antibodies

Antibodies used in this study are listed in Table 3. Preparation and characterization of anti-SOD1^SO3H^, SOD1^int^, and apoSOD antibodies have been described in our previous papers [34–36]. The 24–39 antibody was raised in rabbits immunized with a peptide corresponding to Glu24 – Thr39 in human SOD1 with an additional Cys at its N-terminus (Eurofins Genomics) and affinity-purified with the peptide conjugated with SulfoLink™ Coupling Resin (Thermo Fisher Scientific).

**Table 3:**
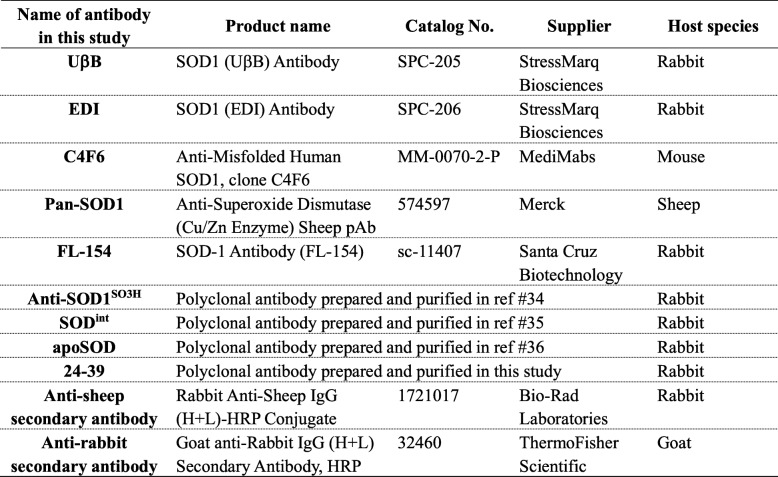
A list of antibodies used in this study

### Preparation of recombinant SOD1 proteins

Recombinant SOD1 proteins were expressed in *E. coli* SHuffle™ (New England Biolabs) and purified as previously reported [7]. A thiol-disulfide status in SOD1 proteins was confirmed by iodoacetamide modification followed by non-reducing SDS-PAGE, which is based upon a retarded electrophoretic mobility of disulfide-reduced SOD1 [37]. SOD1 in the presence of copper and zinc ions was prepared by incubation of an apo form of SOD1 with an equimolar amount of copper and zinc ions at 37 °C for an hour. Disulfide-crosslinked oligomers (S-S oligomers) and insoluble aggregates of SOD1 were prepared as previously described [7, 8].

### Western blotting

CSF and recombinant SOD1 samples were prepared in the Laemmli sample buffer with 6.7% β-mercaptoethanol, separated in 12.5% polyacrylamide gels by SDS-PAGE, and then blotted on PVDF membranes. After the membranes were blocked with 1% (w/v) skim milk in phosphate-buffered saline (PBS) containing 0.05% Tween-20 (PBS-T), the blots were probed with primary antibodies followed by their corresponding secondary antibodies. Detailed conditions for Western blotting are indicated in figure legends.

### In-gel SOD1 activity assay

SOD1 activity in CSF was evaluated with an in-gel assay using nitro blue tetrazolium [38]. CSF (20 μg of proteins) was prepared in a Native-PAGE sample buffer containing 0.1 mM EDTA and then separated with Native-PAGE using a 10% polyacrylamide gel (1.5 mm thickness) in a Native-PAGE running buffer containing 5 mM EDTA on ice. After the electrophoresis, the gel was soaked in 1 g/L nitro blue tetrazolium followed by 100 mM potassium phosphate at pH 7.0 containing 140 μM riboflavin 5′-phosphate sodium and 0.2% (v/v) *N*,*N*,*N*′,*N*′-tetramethyl ethylenediamine in the dark. The gel was then exposed to a light box until achromatic bands representing the SOD1 activity were developed.

### ELISA

Indirect and sandwich ELISAs were performed as previously reported [35, 36]. Briefly, for indirect ELISA, 5 μg of SOD1 proteins in 50 μL were first adsorbed on each well of an ELISA plate, and the wells were blocked with 0.5% (w/v) bovine serum albumin (BSA). The adsorbed SOD1 proteins were then detected with primary antibodies (0.2 μg/mL) followed by the corresponding secondary antibodies (1:1000 dilution). As a substrate solution, *O*-phenylenediamine and 0.012% H_2_O_2_ in a buffer containing 100 mM sodium citrate at pH 5.0 were used. Absorbance was read at 490 nm using a plate reader (Epoch, BioTek) and was called as ELISA signal intensity. The ELISA signal intensities were then divided by those obtained with 5 μg of BSA in 50 μL instead of SOD1 proteins and shown as “ELISA signal (vs. BSA)”.

For sandwich ELISA, capture antibodies (UβB, 1 μg/mL: EDI, 1 μg/mL: apoSOD, 5 μg/mL: 24–39, 2 μg/mL: C4F6, 1:500 dilution: FL-154, 0.2 μg/mL) were first adsorbed on wells of an ELISA plate, to which samples (1.0 μg of recombinant SOD1 proteins: 20 μg of total proteins in CSF samples) were applied. A CSF sample for the sandwich ELISA was prepared by addition of PBS so as to contain 20 μg of total proteins in 100 μL of total volume. To prepare a standard curve, a series of recombinant SOD1 proteins across a range of amounts (0.01 ng – 100 ng in 100 μL) were also examined with sandwich ELISA using the capture antibodies (Additional file 3: Figure S2, absorbance values available in Additional file 9). Equations were obtained by fitting the standard curves (0.01 ng – 10 ng) with exponential functions and shown in the figures (Additional file 3: Figure S2). Given distinct reactivities of the antibodies to different SOD1 variants (Additional file 4: Figure S3, Additional file 6: Figure S5), we used a SOD1 variant with a relatively high value of ELISA signal intensity for the standard curve preparation as well as a positive control in ELISA with each of the antibodies. More specifically, A4V-mutant apo-SOD1^S-S^, G85R-mutant apo-SOD1^S-S^, wild-type apo-SOD1^S-S^, G37R-mutant apo-SOD1^S-S^, and wild-type holo-SOD1^S-S^ were used as positive standards for ELISA with UβB/EDI, apoSOD, 24–39, C4F6, and FL-154 respectively. After vigorous washing of the wells with PBS-T, detection antibodies (Pan-SOD1, 1:500 dilution) followed by the corresponding secondary antibodies were added (anti-sheep secondary antibody, 1:500 dilution). As in indirect ELISA, *O*-phenylenediamine and 0.012% H_2_O_2_ in a buffer containing 100 mM sodium citrate at pH 5.0 were used as a substrate solution, and absorbance was read at 490 nm using a plate reader (Epoch, BioTek). To semi-quantitatively examine the presence of misfolded SOD1 in CSF with our sandwich ELISA, nonetheless, we would just like to mention that distinct SOD1 variants are not necessarily required for the different antibodies as long as absorbance values obtained with the samples were within a range covered in a standard curve with any recombinant SOD1 variants. Also, an important caveat is that the recombinant SOD1 standards are expected to have a distinct reactivity toward the capture antibodies from that of SOD1 in CSF. In other words, substitution of the observed absorbance values into equations fitted to the standard curves would give us an *apparent* amount of misfolded SOD1 in CSF based upon the reactivity toward the capture antibodies. In this study, therefore, we estimated “apparent amounts of SOD1” (or indicated as “SOD1 (ng/20 μg of total CSF proteins)” in axis of our graphs) based upon the observed absorbance values with the equations fitted to the standard curves. Apparent amounts of SOD1 as well as absorbance values were available (Additional file 9).

### Immunoprecipitation of misfolded SOD1 from CSF

Misfolded SOD1 in CSF was absorbed by C4F6-conjugated Dynabeads® M-270 Epox according to the manufacture’s recommendation with slight modifications (Invitrogen). In brief, Dynabeads® M-270 Epox (1 × 10^8^ beads) was first equilibrated in 100 mM sodium phosphate buffer at pH 7.4 and then coupled with either C4F6 (1:50 dilution) or normal mouse IgG (1:50 dilution; Negative control mouse IgG, Dako) in 100 mM sodium phosphate buffer at pH 7.4 containing 1 M ammonium sulfate with slow rotation at 37 °C for 24 h. After the antibody-coated magnetic beads were washed a total of four times with PBS-T, CSF samples (20 or 40 μL) were reacted with the beads with slow rotation at 4 °C for 24 h. The beads were incubated with 100 mM citrate buffer at pH 3.1 to elute misfolded SOD1, and the eluates were analyzed by Western blotting with FL-154 (1:1000 dilution in PBS-T with 5% BSA) followed by the secondary antibody (1:500 dilution in PBS-T; anti-rabbit IgG, HRP-linked antibody, Cell Signaling Technology).

### Viability assay using NSC-34 cell line

A motor neuron-like hybrid cell line, NSC-34, was kindly provided by Professor Neil Cashman (Department of Neurology, the University of British Columbia, Vancouver, Canada) and routinely maintained in a proliferation medium containing Dulbecco’s Modified Eagle Medium (DMEM) with 4.5 g/L D-glucose (nacalai tesque) supplemented with 10% (v/v) fetal bovine serum (FBS) at 37 °C and 5% CO_2_. For cellular differentiation, NSC-34 cells were seeded on a 96-well microplate (1.0 × 10^4^ cells/well) and cultured in the proliferation medium for 24 h. The medium was changed to a differentiation medium containing a 1:1 mixture of DMEM/F12 (Ham) with GlutaMAX™ (Gibco) supplemented with containing 1% (v/v) FBS, 0.1 mM non-essential amino acids, and 10 μM all-*trans* retinoic acid (Wako), and the culture was continued for 48 h. NSC-34 cells were then exposed to 10% (v/v) CSF in the differentiation media without all-*trans* retinoic acid. Each CSF sample was tested in duplicate. As a negative control, 10% (v/v) PBS was used instead of 10% (v/v) CSF. Following 48 h of the CSF exposure, the cells were analyzed for their viability with Cell Counting Kit-8 (Dojindo). The data were represented as the averaged cell viability relative to that of the negative control (i.e. the cells exposed to PBS instead of CSF). For the statistical analysis, a multiple comparison test was performed on twelve groups; namely, CSF, CSF with mouse IgG, and CSF with C4F6 from non-ND, PD^SOD1^/PSP^SOD1^, PD/DLB, and ALS. Because the data sets from some of those twelve groups lacked the normal distribution, the Scheffe’s *F* test was used. For the identification of dying cells, the cells were stained with Cellstain DAPI (1 μg/mL, Dojindo), which only inefficiently pass through an intact cell membrane and therefore preferentially stain dead cells [39], and observed using a phase contrast/fluorescence microscope (BZ-710X, Keyence). Each experiment was performed on the same passage.

### Statistics

Results are given as the mean ± S.D. All statistic tests were performed using a Statcel 4 software (OMS Publishing Inc.). Data sets were first tested whether they exhibited the normal distribution pattern. When the data sets were characterized by the normal distribution, Student’s *t*-test or Welch’s *t-*test was performed for the statistical analysis on the data with or without homogeneity of variance, respectively. For the data sets lacking the normal distribution, the statistical analysis was performed with Mann-Whitney *U*-test.

## Results

### Wild-type SOD1 can be oxidatively modified in CSF of neurodegenerative diseases

As summarized in Tables 1 and 2, CSF samples of neurodegenerative diseases in this study were obtained from twenty-one ALS cases, thirteen PD cases, five PSP cases, four AD cases, and one dementia with Lewy body (DLB) case. Also, seventeen non-neurodegenerative disease (non-ND) cases were examined (Table 1). All ALS cases except ALS10 were sporadic, among which no mutation in exons of *SOD1* gene was further confirmed in ALS1, 2, 3, 4, 6, 7, 8, and 9 (Table 2). ALS10 was a familial ALS case with the C111Y mutation in the *SOD1* gene [40].

**Fig. 1:**
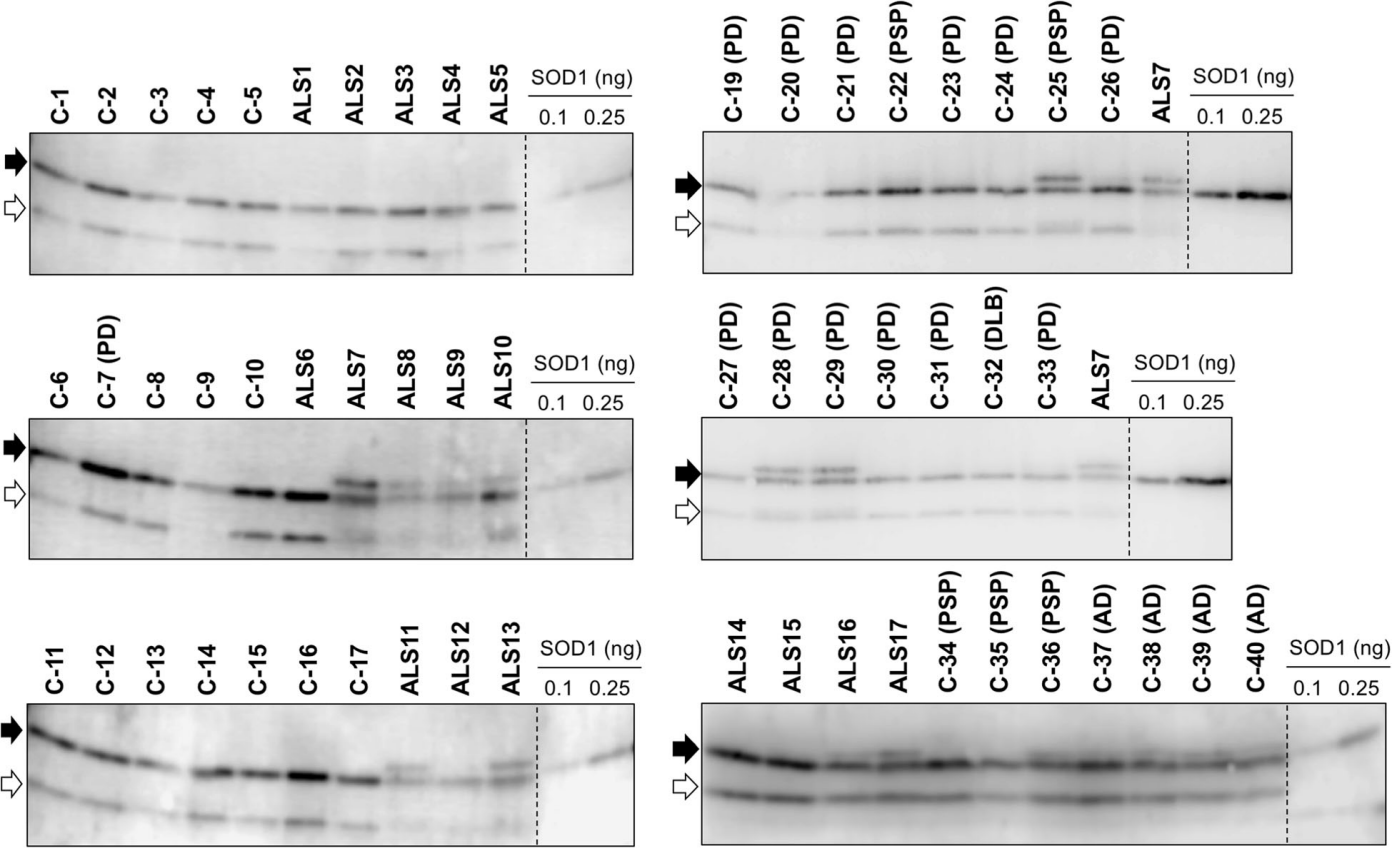
Sulfonylation of wild-type SOD1 in CSF from some neurodegenerative disease cases. CSF samples (4 μg of total proteins) as well as recombinant human SOD1 proteins (0.1 and 0.25 ng) were analyzed by Western blotting with an anti-SOD1 antibody FL-154. The blots on the membranes were first reacted with FL-154 (0.2 μg/mL) in PBS-T with 1% (w/v) skim milk and then probed with anti-rabbit secondary antibody in PBS-T with 0.5% (w/v) skim milk (1:2000 dilution). A major band corresponding to full-length SOD1 is indicated with a filled arrow, while a band with an open arrow shows a truncated form of SOD1

We first attempted to detect SOD1 in those CSF samples by Western blotting using a SOD1 antibody FL-154 (Table 3) and found multiple SOD1-positive bands (Fig. 1). FL-154 is a polyclonal antibody that is raised against a full-length human SOD1 protein. Among those multiple bands, a major band indicated by a filled arrow had the same electrophoretic mobility with that of recombinant wild-type SOD1. A band corresponding to truncated SOD1 was also observed, albeit in different degrees among cases, in both ALS and non-ALS cases (open arrows in Fig. 1). This is consistent with a previous study that suggested no pathological roles of truncated SOD1 in ALS [25].

**Fig. 2:**
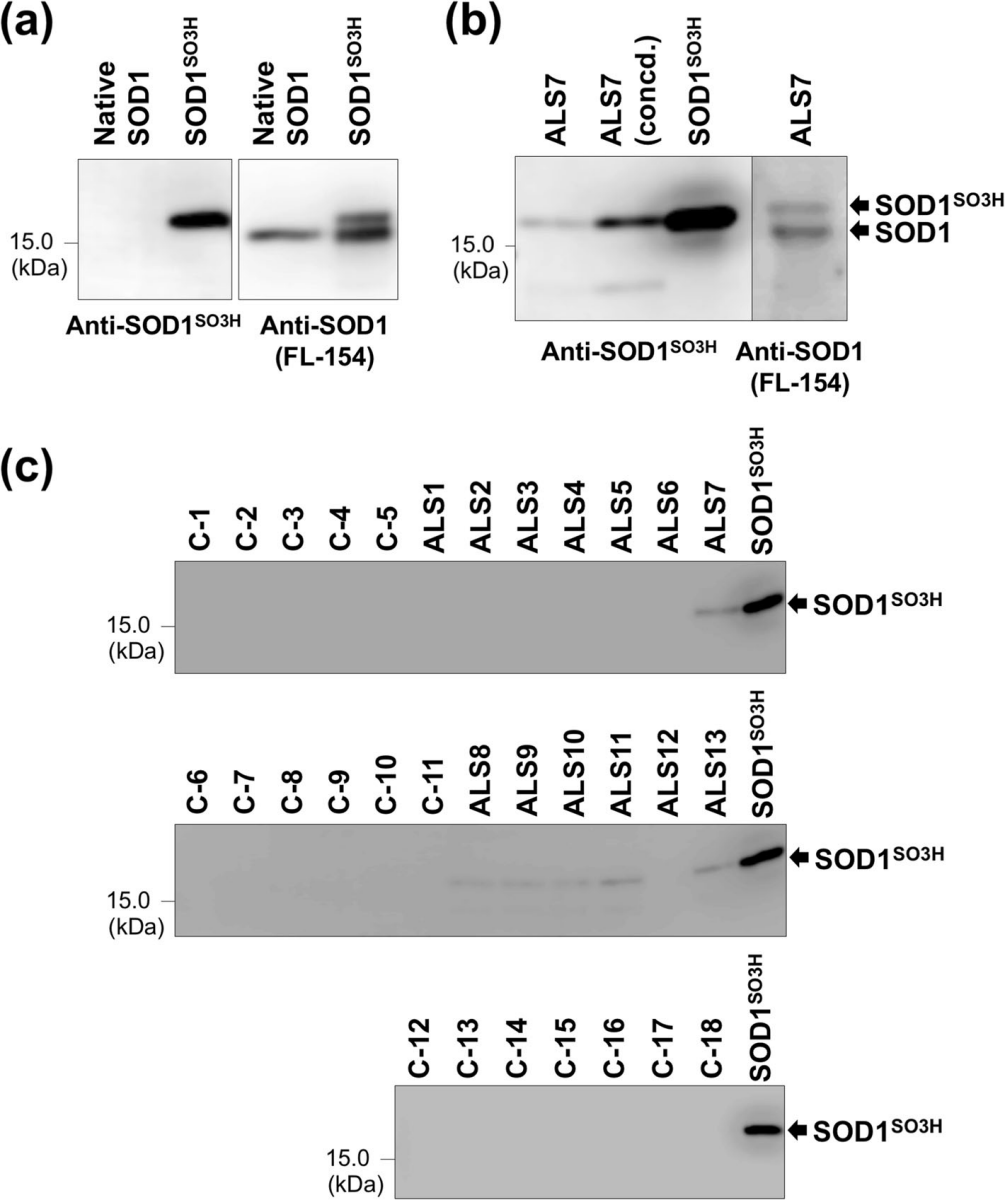
Detection of SOD1^SO3H^ in CSF of ALS cases using anti-SOD1^SO3H^ antibody. (**a**) Native SOD1 (Sigma, #S9636: 20 μU) and a recombinant sample containing SOD1^SO3H^ (2.5 ng) were analyzed by Western blotting side-by-side with (left) anti-SOD1^SO3H^ and (right) FL-154 antibodies. The recombinant sample containing SOD1^SO3H^ produced doublet bands in the Western blot with FL-154 (right), but only the upper band was recognized with anti-SOD1^SO3H^ antibody (left). Also, native SOD1 without any oxidation was detected as a single band in the Western blot with FL-154 (right) but was not recognized with anti-SOD1^SO3H^ antibody (left). These results confirm the retarded electrophoretic mobility of SOD1 upon sulfonylation of Cys and also support the specificity of our anti-SOD1^SO3H^ antibody toward SOD1^SO3H^. (**b**) The CSF sample from ALS7 (4 μg of total proteins; ALS7 concd. Sample contained 10 μg of total proteins) was analyzed by Western blotting side-by-side with (left) anti-SOD1^SO3H^ and (right) FL-154 antibodies. Doublet SOD1-positive bands were observed in the Western blot probed with FL-154 (right), among which only the upper band was recognized with anti-SOD1^SO3H^ antibody and also exhibited the same electrophoretic mobility with that of recombinant SOD1^SO3H^ (20 ng). (**c**) CSF samples (4 μg of total proteins) and recombinant SOD1^SO3H^ (10 ng) were analyzed by Western blotting with anti-SOD1^SO3H^ antibodies. In all panels, the blots on the membranes were reacted with either FL-154 (0.2 μg/mL) in PBS-T with 1% (w/v) skim milk or anti-SOD1^SO3H^ (0.06 μg/mL) in PBS-T with 3% (w/v) BSA, and then probed with anti-rabbit secondary antibody in PBS-T with 0.5% (w/v) skim milk (1:2000 dilution)

Notably, in a subset of the neurodegenerative disease cases including ALS, PD, PSP, and AD, an additional band was observed with slightly less mobility than that of the major band shown by the filled arrows (Fig. 1). Previous studies have shown that glutathionylation at Cys111 of SOD1 in vivo could retard the electrophoretic mobility of SOD1 [41]; however, the samples were electrophoresed in the presence of a reductant, β-mercaptoethanol, where the glutathione group, if any, at Cys111 should be removed. Alternatively, the additional upper band is reminiscent of SOD1 sulfonylated at Cys111 in vitro (called SOD1^SO3H^) [34]; indeed, we confirmed that the additional upper band in the CSF samples exhibited the same electrophoretic mobility with that of recombinant SOD1^SO3H^ and was detected with our anti-SOD1^SO3H^ antibody (Fig. 2). It is also important to note that the additional upper band was observed in ALS10 with the heterozygous C111Y mutation in the *SOD1* gene (Fig. 1). Given that the position at 111 in C111Y-mutant SOD1 is no longer available for sulfonylation, these results suggest that wild-type SOD1 can be sulfonylated in CSF of the *SOD1*-ALS as well as sALS cases without *SOD1* mutations. Nonetheless, not all of the ALS cases were characterized by SOD1^SO3H^ in their CSF, indicating that SOD1^SO3H^ is not a pathological hallmark of ALS. SOD1^SO3H^ was also detected in some cases of the other neurodegenerative diseases (PD, PSP, AD) but not in all of the non-ND cases (Fig. 1). Therefore, certain pathological changes caused by neurodegeneration might facilitate the sulfonylation at the Cys residue of SOD1 in CSF.

### Significant fractions of wild-type SOD1 are misfolded in CSF of ALS

**Fig. 3:**
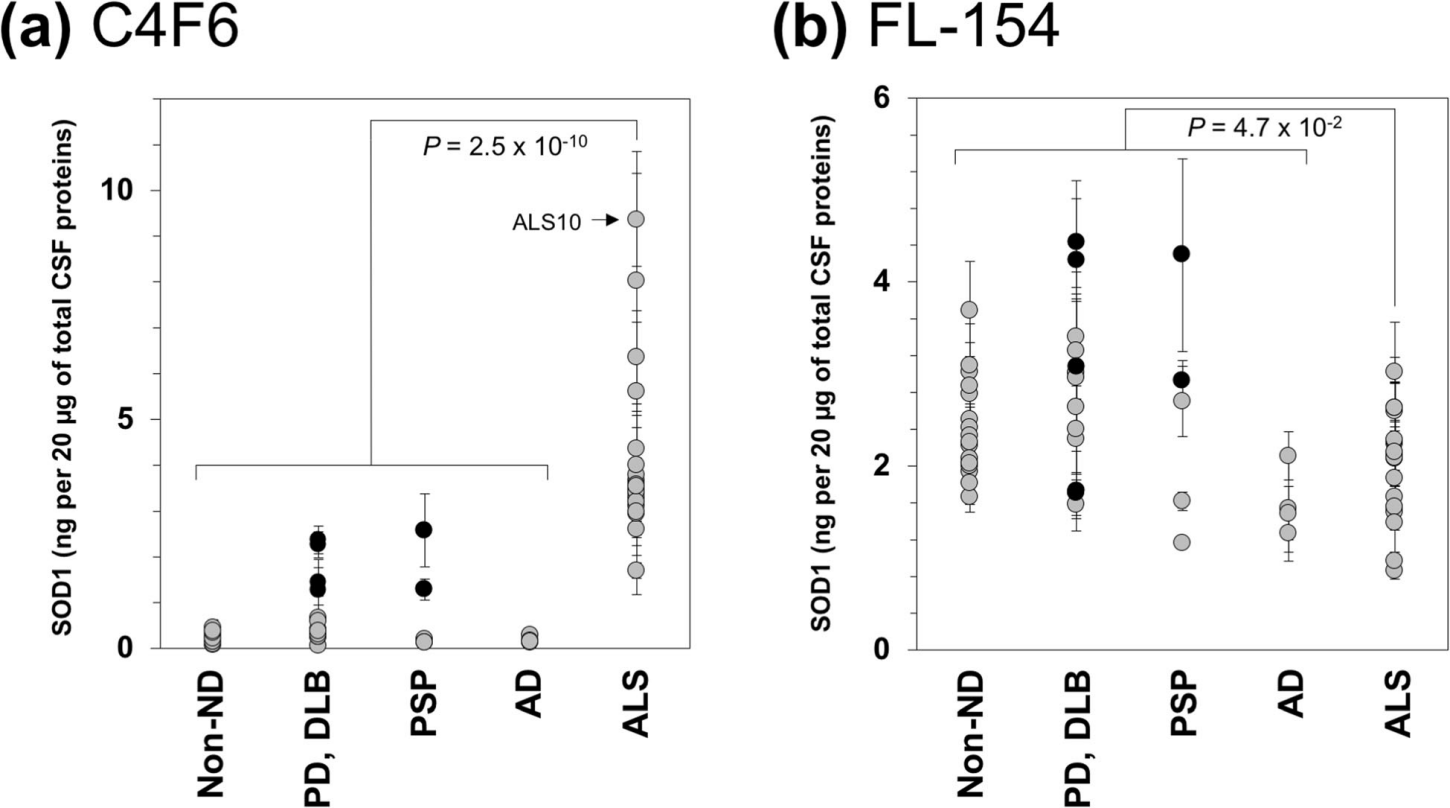
CSF of ALS cases contains the species recognized by misfolded SOD1-specific antibody C4F6. The CSF samples (20 μg of total proteins, 100 μL of volume) from Non-ND, PD/DLB, PSP, AD, and ALS cases were analyzed by a sandwich ELISA with (**a**) C4F6 and (**b**) FL-154 as capture antibodies, and Pan-SOD1 was used as a detection antibody. As described in the Methods, absorbance values at 490 nm measured in the ELISA were substituted into equations fitted to standard curves (Additional file 3: Figure S2), and apparent amounts of SOD1 were thereby estimated and plotted as SOD1 (ng/20 μg of total CSF proteins). The data on the PD and PSP cases containing the C4F6-reactive SOD1 (also see Fig. 4) were shown in black filled circles. Three independent experiments were performed to estimate error bars (standard deviation). The statistical differences were analyzed with Mann-Whitney *U*-test, and *P* values are shown

In order to test if SOD1 was misfolded in CSF of the ALS cases, a sandwich ELISA was performed by using a misfolded SOD1-specific antibody C4F6 [42]. C4F6 is a monoclonal antibody that has been generated by using recombinant SOD1 with G93A mutation, and we confirmed the selectivity of C4F6 toward misfolded/mutant SOD1 over wild-type SOD1 (Additional file 4: Figure S3). Also, an “apparent” amount of SOD1 was estimated by substitution of the observed absorbance values in the ELISA into an equation fitted to a standard curve (Additional file 3: Figure S2), which was described in detail in the Methods (data sets available in Additional file 9). Given that conformational information on misfolded SOD1, if any, in CSF is not available, we would like to emphasize that our recombinant SOD1s as calibrants are not faithful models of the misfolded SOD1 in CSF and that amounts of SOD1 calculated from our standard curves will be apparent. As shown in Fig. 3a, it is evident that apparent amounts of C4F6-reactive SOD1 were significantly higher in the ALS cases compared to those in the non-ALS cases including non-ND, PD, DLB, PSP, and AD (*P* = 2.5 × 10^− 10^, Mann-Whitney *U*-test) (Fig. 3a). ALS10 having the C111Y mutation in the *SOD1* gene exhibited the highest amounts of C4F6-reactive SOD1, implying the mutation-induced misfolding of SOD1. Between sALS (the ALS cases except ALS10) and non-ALS, nonetheless, statistically significant differences still remained in amounts of C4F6-reactive SOD1 (*P* = 4.8 × 10^− 10^, Mann-Whitney *U*-test). When a conformationally non-selective SOD1 antibody, FL-154, was used as a capture antibody for the sandwich ELISA, we found that apparent amounts of SOD1 in ALS were slightly fewer than those of non-ALS; however, the differences were less significant (*P* = 4.7 × 10^− 2^, Mann-Whitney *U*-test) (Fig. 3b). These data thus show that all ALS cases examined here contain the C4F6-reactive misfolded form(s) of wild-type SOD1 in CSF.

**Fig. 4:**
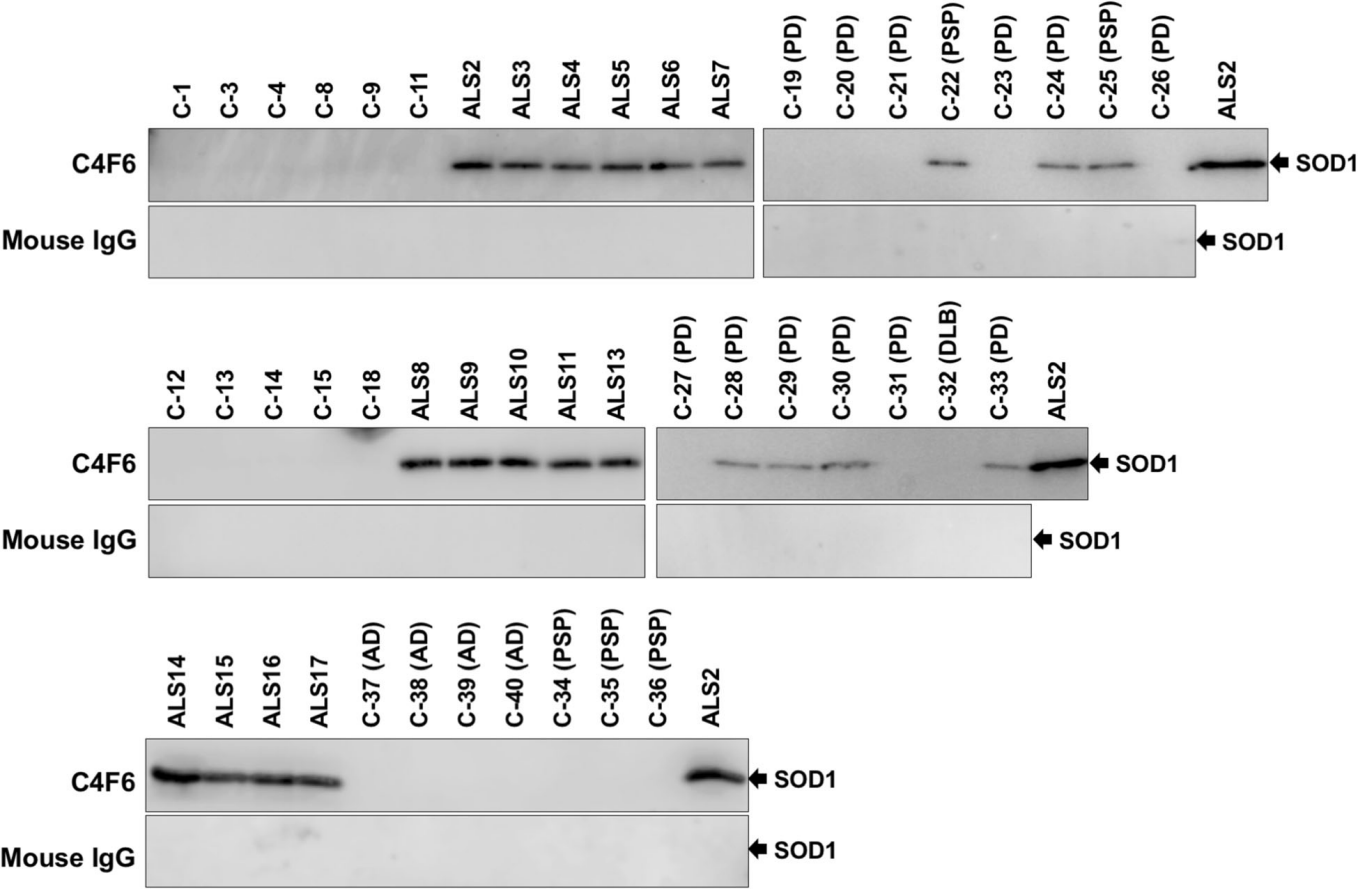
Immunoprecipitation experiments confirm the presence of C4F6-reactive SOD1 in CSF of all ALS cases as well as some of PD and PSP cases. The C4F6-crosslinked or mouse IgG-crosslinked magnetic beads were first incubated with CSF (20 μL) at 4 °C for 24 h, and the SOD1 proteins bound to those magnetic beads were then eluted with 10 μL of 100 mM citrate buffer at pH 3.1. The eluates (10 μL) were analyzed by Western blotting with FL-154 antibody. Due to the limited availability of the CSF samples, we could not examine several cases including C-2, C-5, C-6, C-7, C-10, C-16, C-17, ALS1, ALS12, ALS18, ALS19, ALS20, and ALS21

To further confirm the presence of the C4F6-reactive, misfolded SOD1 in CSF of the ALS cases, immunoprecipitation experiments with C4F6 were performed. For that purpose, magnetic beads (Dynabeads® M-270 Epox) were first conjugated with C4F6 and then applied to the CSF samples. The immunoprecipitates were analyzed with Western blotting, showing that SOD1 proteins were indeed isolated from the CSF samples of all ALS cases examined but not from those of the non-ND cases (Fig. 4). Unexpectedly, the C4F6-reactive misfolded SOD1 was also detected in some PD cases (5 out of 12 cases: C-24, C-28, C-29, C-30, and C-33) as well as two PSP cases (C-22 and C-25) albeit with significantly smaller amounts (Fig. 4). When re-examined, our sandwich ELISA with C4F6 showed that those PD and PSP cases with the C4F6-reactive misfolded SOD1 (called PD^SOD1^ and PSP^SOD1^ cases, respectively) were characterized by slightly higher apparent amounts of C4F6-reactive SOD1 than those in the cases without it (shown as black filled circles in Fig. 3a). Also notably, neither the truncated SOD1 nor the sulfonylated SOD1 was immunoprecipitated with the C4F6-conjugated magnetic beads (Fig. 4), suggesting no significant roles of truncation/sulfonylation in the formation of the C4F6-reactive SOD1 in CSF. When a normal mouse IgG was used as an immunoprecipitant, furthermore, no SOD1 proteins were detected in all cases examined here (Fig. 4). Based upon these results, the ALS cases are characterized by the C4F6-reactive misfolded SOD1 in CSF, which would have pathological roles also in a subset of the PD and PSP cases.

**Fig. 5:**
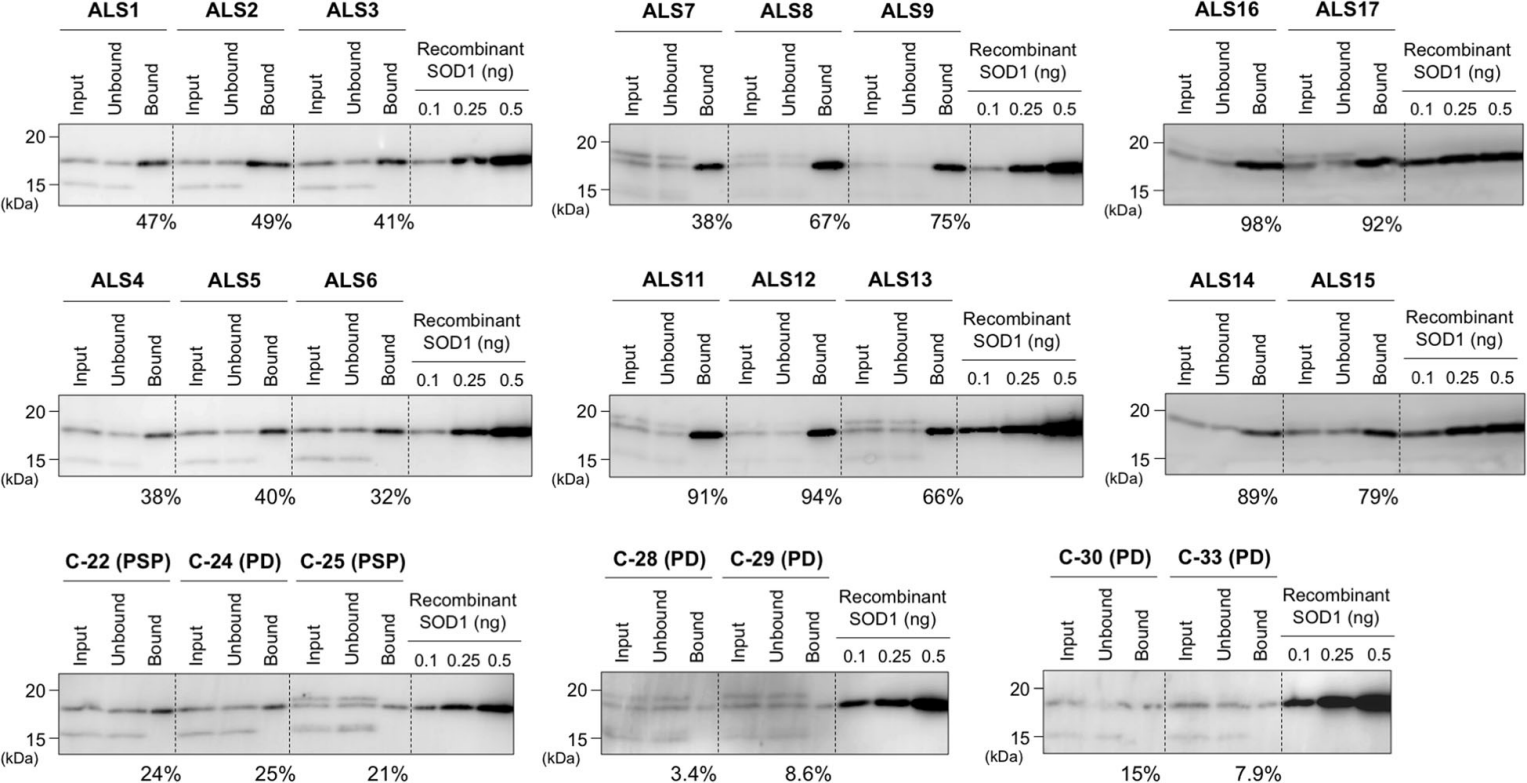
Most of SOD1 in CSF from ALS were recognized by misfolded SOD1-specific antibody C4F6. C4F6-crosslinked magnetic beads were first incubated with CSF (40 μL) at 4 °C for 24 h, and the solutions were collected as the “unbound” fraction. The remaining magnetic beads were then treated with 10 μL of 100 mM citrate buffer at pH 3.1 in order to elute SOD1 proteins captured by the magnetic beads (the “bound” fraction). Ten microliter of CSF (input), the unbound, and the bound fractions were analyzed by Western blotting with FL-154 antibody. The percentage of C4F6-reactive SOD1 in the CSF samples was roughly estimated by measuring the band intensity of SOD1 in the unbound and bound fractions and indicated at the bottom of each gel. Details were described in the Methods

To roughly estimate amounts of the misfolded SOD1 in CSF of the ALS, PD^SOD1^, and PSP^SOD1^ cases, the CSF samples were separated into fractions that were bound and not bound to the C4F6-conjugated magnetic beads, and the SOD1-immunoreactive bands in the Western blots of those fractions were then calibrated with those of recombinant SOD1 proteins in fixed amounts. As summarized in Fig. 5, significant amounts of the C4F6-reactive misfolded SOD1 were detected in the bound fraction (immunoprecipitate) and roughly estimated as 30–90% of total SOD1 proteins in CSF of the ALS cases. When the unbound fraction was further treated with the C4F6-conjugated magnetic beads, SOD1 proteins were no longer immunoprecipitated (Additional file 5: Figure S4), confirming that SOD1 in the unbound fraction was not recognized by C4F6. In the PD^SOD1^ and PSP^SOD1^ cases, moreover, the percentage of the immunoprecipitated SOD1 with C4F6 was much less (< 30% of total SOD1) than that in the ALS cases (Fig. 5). Taken together, we found for the first time that most of the SOD1 proteins in CSF of the ALS cases were misfolded.

### Misfolded conformation(s) of pathological wild-type SOD1 in CSF of ALS

To get insights into the misfolded conformation(s) of wild-type SOD1 in CSF of the ALS cases, several other polyclonal antibodies that recognize distinct epitopes only available in misfolded SOD1 were used as capture antibodies in the sandwich ELISA; apoSOD [36], EDI, UβB, 24–39 [27], and SOD1^int^ [35] (Table 3). As described later, apoSOD, 24–39, and SOD1^int^ have been known to recognize Thr54 – His63, Glu24 – Thr39 and His48 – Asn53 as an epitope, respectively. Also, EDI and UβB are commercially available antibodies that are prepared by using the same antigens with those in SEDI [43] and USOD [18] antibodies (R^143^LACGVIGI^151^ and L^42^HGFHVH^48^), respectively. The conformation-dependent specificity of those polyclonal antibodies was first confirmed using recombinant wild-type and ALS-mutant (A4V, G37R, and G85R) SOD1 proteins, which differed in the presence/absence of copper and zinc ions, a thiol-disulfide status, and oligomerized/aggregated states. Toward these various states of SOD1, the antibodies exhibited distinct reactivities in an ELISA, a part of which was previously published from our group [35, 36], and wild-type SOD1 in the presence of copper and zinc ions as well as the disulfide bond was the least reactive to the misfolded SOD1-specific antibodies (Additional file 6: Figure S5A-E). In contrast, a Pan-SOD1 polyclonal antibody that was raised against SOD1 from human erythrocytes (Table 3) exhibited almost equal reactivities toward all states of SOD1 examined (Additional file 6: Figure S5F). These results thus endorse the specificity of the polyclonal antibodies toward non-native, misfolded conformations of SOD1 proteins.

**Fig. 6:**
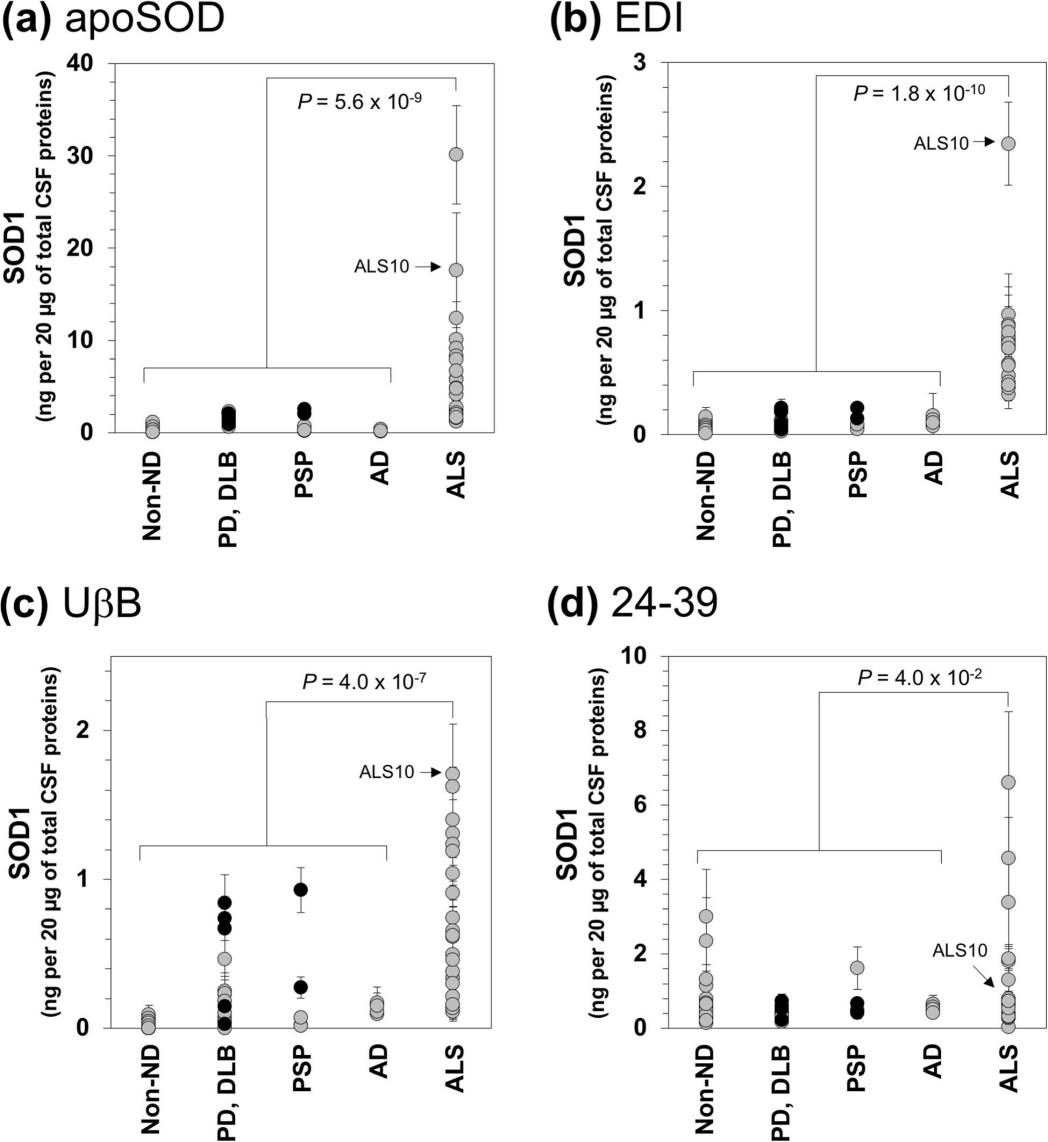
CSF of ALS cases contains the SOD1 species recognized with several misfolded SOD1-specific antibodies. CSF samples (20 μg of total proteins, 100 μL of volume) were analyzed by sandwich ELISA with (**a**) apoSOD, (**b**) EDI, (**c**) UβB, or (**d**) 24–39 as a capture antibody. For all cases, Pan-SOD1 antibody was used as a detection antibody. As described in the Methods, absorbance values at 490 nm measured in the ELISA were substituted into equations fitted to standard curves (Additional file 3: Figure S2), and apparent amounts of SOD1 were thereby estimated and plotted as SOD1 (ng/20 μg of total CSF proteins). The data on PD and PSP cases with C4F6-reactive SOD1 were shown as black filled circles. Three independent experiments were performed to estimate error bars (standard deviation). The statistical differences were analyzed with Mann-Whitney *U*-test, and *P* values are shown in each panel

We then examined the CSF samples with the sandwich ELISA using a panel of those misfolded SOD1-specific antibodies. Figures 6a and b show that the CSF samples of the ALS cases contained significantly higher apparent amounts of SOD1 reactive to apoSOD and EDI antibodies than those of the non-ND and the other neurodegenerative disease cases (PD, DLB, PSP, and AD). As observed in the ELISA results with C4F6 (Fig. 3a), ALS10 with the C111Y mutation in the *SOD1* gene were characterized with relatively high apparent amounts of SOD1 reactive to apoSOD and EDI among the ALS cases, again implying the mutation-induced misfolding of SOD1 (Figs. 6a and b). Even without the data on ALS10, statistically significant differences still existed in the apparent amounts of apoSOD/EDI-reactive SOD1 between sALS and non-ALS cases (*P* values with Mann-Whitney *U*-test are as follows; 1.1 × 10^− 8^ and 3.6 × 10^− 10^ in apoSOD and EDI, respectively). Amounts of apoSOD/EDI-reactive SOD1 in the PD and PSP cases appeared not to be significantly dependent upon the presence and absence of the C4F6-reactive misfolded SOD1 (black filled circles in Fig. 6a and b); this would be partly because an amount of the misfolded SOD1 in PD^SOD1^/PSP^SOD1^ was much less than that of ALS (Fig. 4). The UβB antibody also detected higher amounts of SOD1 in CSF of the ALS cases than those of the non-ALS cases (Fig. 6c), while the differences between the ALS and non-ALS cases were smaller than those obtained in the ELISA with apoSOD and EDI (Figs. 6a and b). Also, values of the data in the PD/DLB and PSP cases were relatively scattered. Nonetheless, the PD^SOD1^ and PSP^SOD1^ cases appeared to show higher reactivities toward UβB than those of the other cases lacking the C4F6-reactive SOD1. In contrast, differences in the apparent amounts of SOD1 reactive to 24–39 antibody were less significant between ALS and non-ALS cases (*P* = 4.0 × 10^− 2^, Mann-Whitney *U*-test; Fig. 6d), which is consistent with previous results reported in [27]. Furthermore, no absorption at 490 nm in our sandwich ELISA with SOD1^int^, which can specifically recognize the disulfide-crosslinked SOD1 oligomers (Additional file 6: Figure S5C) [35], were observed in the CSF samples of both ALS and non-ALS cases (data not shown). Accordingly, SOD1 in CSF of the ALS cases is not randomly misfolded (or unfolded) but appears to assume an abnormal conformation(s) that is definable with distinct reactivities of the conformation-dependent antibodies.

**Fig. 7:**
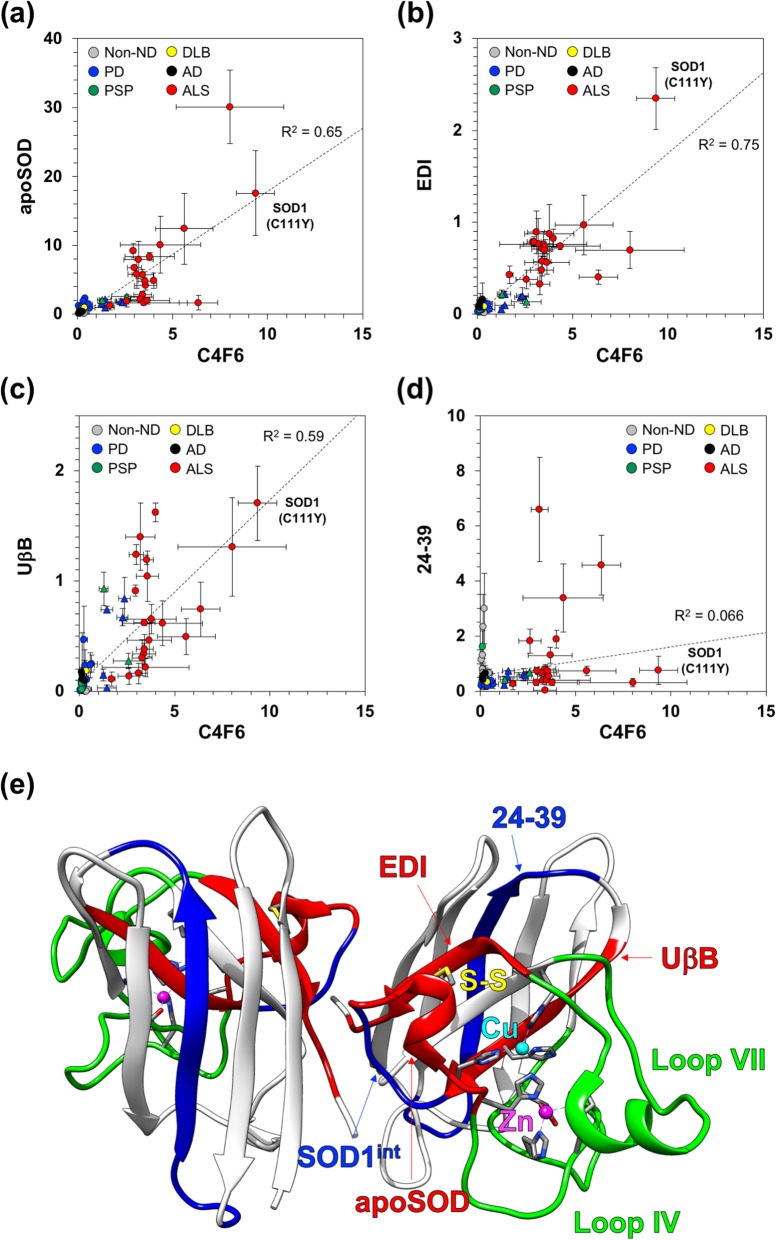
Correlation in apparent amounts of SOD1 was detected with a distinct set of misfolded SOD1-specific antibodies. Apparent amounts of SOD1 in CSF detected with (**a**) apoSOD, (**b**) EDI, (**c**) UβB, and (**d**) 24–39 (Fig. 6) were plotted against those of C4F6 (Fig. 3a). The data are represented as follows: gray circles for non-ND; blue circles for PD; green circles for PSP; a yellow circle for DLB; black circles for AD; red circles for ALS. The data of the PD^SOD1^ and PSP^SOD1^ are shown as triangles. In each panel, linear least-squares fitting to the data was performed, and the best fitting line is indicated (broken line) with a value of R^2^. (**e**) A map of epitopes recognized by the antibodies for the misfolded SOD1 is shown on a crystal structure of a native, enzymatically active form of the SOD1 homodimer (PDB ID: 2C9V). A copper ion (Cu, cyan), a zinc ion (Zn, pink), and a conserved disulfide bond (S-S, yellow) are shown. The epitopes of EDI, UβB, and apoSOD are colored red, while those of 24–39 and SOD1^int^ are colored blue. Loops IV and VII are shown green

Indeed, apparent amounts of SOD1 reactive to apoSOD, EDI, and UβB showed good linear correlations with those of C4F6 (*R*^2^ = 0.65, 0.75, and 0.59, respectively; Fig. 7a-c); in the plots, the data points on the non-ALS cases lacking the C4F6-reactive SOD1 were concentrated near the origin of the coordinates, while those on the ALS cases as well as the PD^SOD1^ and PSP^SOD1^ cases deviated from the origin with the highest intensity in ALS10 with the C111Y mutation in the *SOD1* gene. In contrast, a clear correlation was not confirmed in the plot of the apparent SOD1 amounts between 24-39 and C4F6 (*R*^2^ = 0.066, Fig. 7d). These results would hence support the presence of SOD1 with a certain misfolded conformation(s) that exhibits a similar degree of the reactivities with the four antibodies, C4F6, apoSOD, EDI, and UβB.

To obtain further insights into the non-native conformation(s) of SOD1 in CSF of the ALS cases, epitopes of the SOD1 antibodies used in this study were compared (Fig. 7e). EDI, UβB, and apoSOD, which detected pathological SOD1 in CSF, recognize linear epitopes of Arg143 – Ile151, Leu42 – His48, and Thr54 – His63 in SOD1, respectively (colored red in Fig. 7e) [18, 36, 43]. In the native conformation of SOD1, those epitopes appear to be placed in a protein interior that is covered with two large loops, Loops IV and VII (colored green in Fig. 7e). C4F6 was raised against human SOD1 with the G93A substitution [42] and has been reported to recognize a linear epitope containing amino acids from Asp90 to Asp96 that exhibits higher reactivity to misfolded SOD1 than that of the natively folded SOD1 [20, 44]. Exposure of the C4F6 epitope has been also shown to be modulated by Loops IV and VII [44]. In contrast, the epitope regions of 24–39 and SOD1^int^ antibodies, which did not discriminate the pathological SOD1 species in CSF, correspond to Glu24 – Thr39 and His48 – Asn53, respectively [35] (colored blue in Fig. 7e), and appear to be less affected by conformational changes of Loops IV and VII. Pathological SOD1 in CSF of the ALS cases is thus expected to assume a misfolded conformation(s) where the protein interior covered by Loops IV and VII is exposed.

### Decreased metal-affinity of misfolded SOD1 in CSF of ALS

**Fig. 8:**
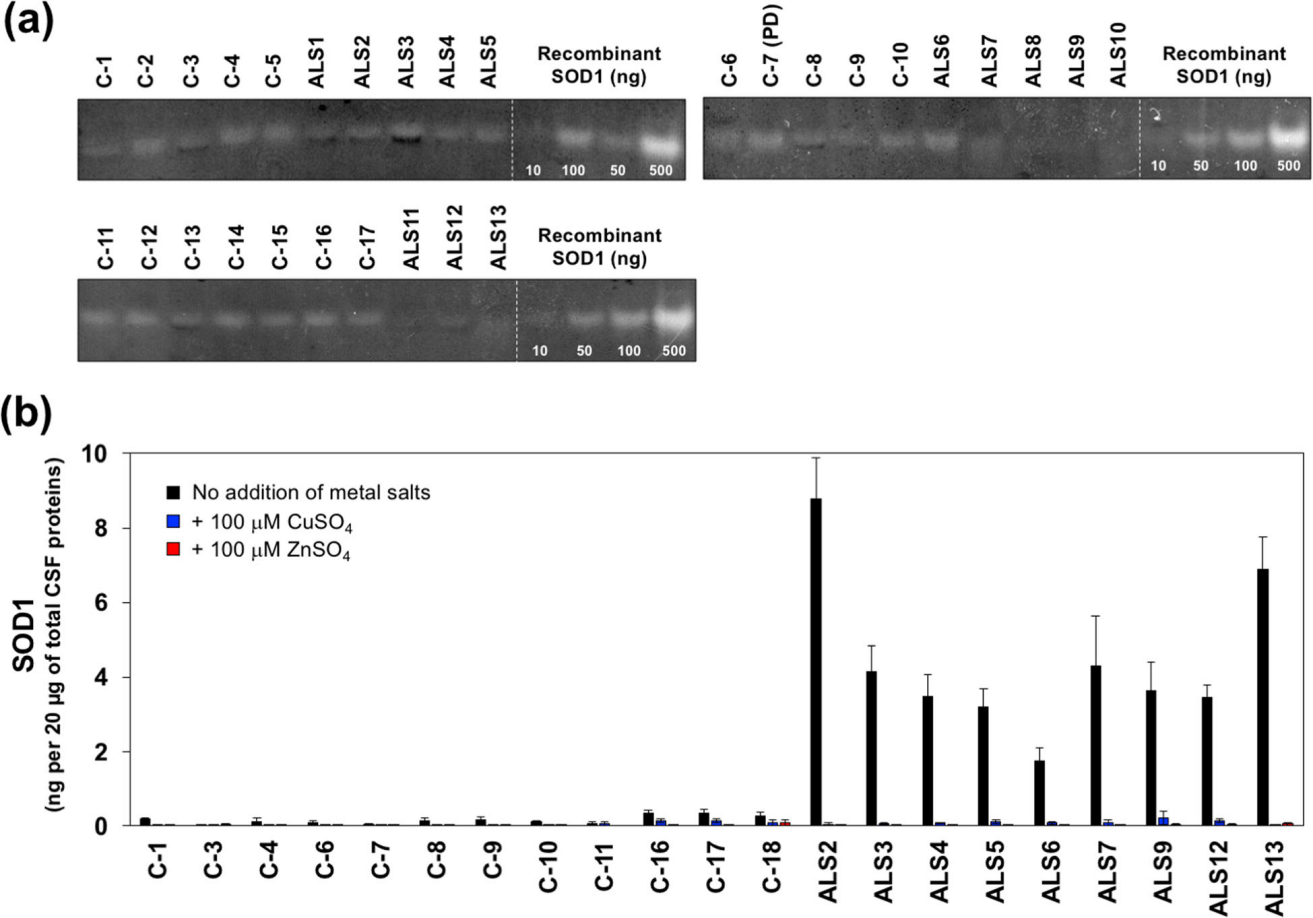
Misfolded SOD1 in CSF of ALS decreases the affinity toward metal ions. (**a**) The CSF samples (20 μg of total proteins) were approximately 4-fold concentrated with a centrifugal filter device and then examined for the SOD1 activities with the in-gel assay. Recombinant wild-type SOD1 protein in the holo state was analyzed as a positive control. (**b**) The CSF samples (20 μg of total proteins, 100 μL of total volume) were first incubated with either (blue bars) 100 μM CuSO_4_ or (red bars) 100 μM ZnSO_4_ at 4 °C overnight and then analyzed by sandwich ELISA with C4F6 and Pan-SOD1 as capture and detection antibodies, respectively. As a control, the CSF samples incubated at 4 °C overnight without addition of any metal ions (black bars) were also examined. As described in the Methods, absorbance values at 490 nm measured in the ELISA were substituted into equations fitted to standard curves (Additional file 3: Figure S2), and apparent amounts of SOD1 were thereby estimated and plotted as SOD1 (ng/20 μg of total CSF proteins). The experiments were performed in triplicate to estimate error bars (standard deviation)

To further characterize the pathological SOD1, we noted that apoSOD antibody detected SOD1 in the CSF samples of the ALS cases (Fig. 6a). This is because our apoSOD antibody can quite exclusively recognize a copper-deficient state of mutant but not wild-type SOD1 in vitro (Additional file 6: Figure S5D) [36]. The native SOD1, which has been known as one of the most stable proteins (*T*_m_, > 90 °C), is required to bind copper and zinc ions and also form the conserved intramolecular disulfide bond for its structural stability [45, 46] as well as enzymatic activity (e.g. [47]). While we confirmed that SOD1 proteins in CSF of ALS and non-ALS controls were equipped with the intramolecular disulfide bond (data not shown), SOD1 activities in CSF appears to be weaker in the ALS than those in the non-ALS cases (Fig. 8a). Also, a percentage of C4F6-reactive SOD1 in CSF of the ALS cases (Fig. 5) appears to be inversely correlated with the degree of SOD1 activity. More precisely, as shown in Figs. 5 and 8a, the ALS cases with a significantly high percentage (> 60%) of C4F6-reactive SOD1 (ALS8, 9, 11, 12, 13) exhibited almost no enzymatic activity of SOD1, while distinct activity bands were observed in the cases with a moderate percentage (< 60%) of C4F6-reactive SOD1 (ALS1, 2, 3, 4, 5, 6). Nonetheless, amounts of SOD1 in CSF were so minute (*approx*. 0.1 ng per 1 μg of total proteins in CSFs, Fig. 1) that the activity of SOD1 was difficult to be quantitatively estimated with the in-gel assay even after 4-fold concentration of the CSF samples by ultrafiltration. Therefore, the apparent inverse correlation between the enzymatic activity and the level of C4F6-reactive SOD1 in CSF needs to be further tested in the future.

Alternatively, apparent amounts of C4F6-reactive SOD1 in CSF of ALS were significantly decreased by pre-incubation of the CSF samples with either 100 μM CuSO_4_ or 100 μM ZnSO_4_ (Fig. 8b), implying the metal-deficiency of SOD1 in CSF of ALS cases. When a polyclonal anti-SOD1 antibody, FL-154, was used as a capture antibody, apparent amounts of SOD1 estimated with ELISA were not significantly affected by incubation of the CSF samples with CuSO_4_ and ZnSO_4_ (Additional file 7: Figure S6), confirming the retention of SOD1 in the CSF samples incubated with the metal ions. CSF contains a significantly small concentration of SOD1 (around 5 nM based upon the densitometric analysis on Fig. 1), and also, SOD1 can bind Cu/Zn ions very tightly (*K*_d_: 6.0 × 10^− 18^ for Cu, 4.2 × 10^− 14^ for Zn) [48]. Therefore, addition of either 10 μM CuSO_4_ or 10 μM ZnSO_4_ to the CSF samples should be sufficient to eliminate the C4F6-reactive SOD1. This was, however, not the case; even after supplemented with either 10 μM CuSO_4_ or 10 μM ZnSO_4_, the CSF samples from ALS cases were found to contain significant amounts of C4F6-reactive SOD1 in our sandwich ELISA (data not shown). Higher levels of copper and zinc in CSF have been reported in ALS cases than those in non-ALS cases [49–51]; therefore, we suppose that SOD1 in CSF of ALS is not an apo state simply lacking the metal ions. As described above, the apo state of wild-type SOD1 is not recognized by our apoSOD (Additional file 6: Figure S5D), but a fraction of SOD1 proteins in CSF of ALS was detected with apoSOD in our sandwich ELISA (Fig. 6a). SOD1 in CSF of ALS is hence considered to assume an abnormally misfolded conformation with significantly reduced affinity toward the metal ions.

An important precedent can be found in CSF of PD cases, where the activity of a copper-dependent enzyme, ceruloplasmin (Cp), was shown to decrease in spite of the increased copper levels [52]. This is partly because copper-induced oxidative modifications in Cp decrease the affinity toward copper ions [53]. CSF from ALS patients have been found to become more oxidizing in the disease progression [54], and increased oxidative stress has been proposed as a factor that can misfold SOD1. Upon treatment with hydrogen peroxide in vitro, for example, wild-type SOD1 was shown to decrease the affinity toward copper/zinc ions [55], and form toxic oligomers/aggregates [10, 23, 29]. In particular, oxidation at the His residues ligating Cu/Zn ions is well expected to significantly reduce an affinity of SOD1 toward metal ions [56]. While possible oxidation of SOD1 in CSF of ALS remains to be investigated in more detail, increased levels of oxidative stress in CSF of ALS cases would have roles in pathological changes of SOD1 through abnormal modification(s) that weakens an affinity of SOD1 to the metal ions.

### CSF exhibits misfolded SOD1-dependent toxicity toward motor neuron-like cells

**Fig. 9:**
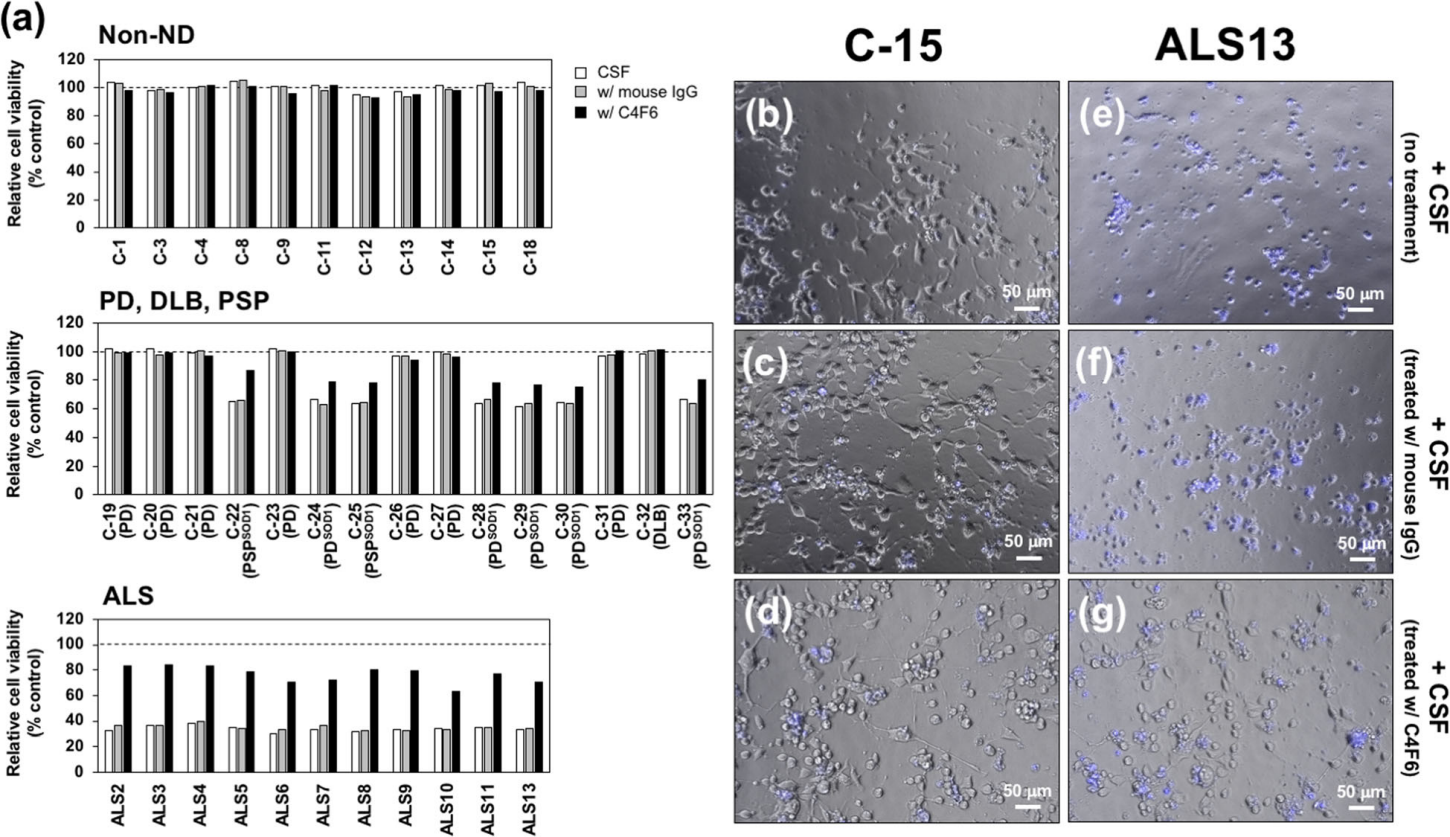
Toxicity of CSF samples from sALS toward motor neuronal cell line comes from misfolded SOD1. (**a**) Differentiated NSC-34 cells were exposed to (white bars) the CSF samples, (gray bars) the CSF samples preabsorbed with normal mouse IgG, and (black bars) the CSF samples preabsorbed with mouse monoclonal C4F6 antibody for 48 h, and the cell viability was assayed with Cell Counting Kit-8. Each of the CSF samples was tested in duplicate, and the data are shown as the averaged cell viability relative to that of the negative control, in which PBS instead of CSF samples was exposed to the cells. (**b**-**g**) Representative images of the differentiated NSC-34 cells exposed to the CSF samples from (**b**-**d**) non-ND (C-15) and (**e**-**g**) sALS (ALS13) are shown. The cells were incubated with (**b**, **e**) the CSF samples, (**c**, **f**) the CSF samples preabsorbed with mouse IgG, and (**d**, **g**) the CSF samples preabsorbed with mouse monoclonal C4F6 antibody. The dying cells were stained with DAPI (shown in blue)

CSF from sALS patients have been known to exhibit toxicity toward motor neuron-like cells NSC-34 [28] albeit with some controversies on NSC-34 cells as a model of motor neurons [57], but it remains unclear whether any SOD1 abnormalities in CSF are involved in their toxicity. As confirmed in our current study, the CSF samples from our ALS cases caused almost 70% reduction in viability of differentiated NSC-34 cells, but the viability was little affected by addition of the CSF samples from non-ND cases (shown as white bars in Fig. 9a; *P* < 0.01, ALS vs. non-ND). Notably, moderate decrease (*approx.* 40% reduction) in the viability was observed in the PD^SOD1^ and PSP^SOD1^ cases (*P* < 0.01 in PD^SOD1^/PSP^SOD1^ vs. non-ND and also vs. ALS) but not in the PD cases without the misfolded SOD1 (*P* > 0.05 in PD/DLB vs. non-ND) (Fig. 9a, white bars). Given that amounts of the misfolded SOD1 were less in the PD^SOD1^ and PSP^SOD1^ cases than in the ALS cases (Fig. 4), the toxicity of the CSF samples toward NSC-34 cells hence appears to be correlated with amounts of the misfolded SOD1.

As shown in Fig. 8b, addition of CuSO_4_/ZnSO_4_ to the CSF samples in large excess (*approx.* 100 μM CuSO_4_/ZnSO_4_ to *approx.* 5 nM SOD1 in CSFs) decreased apparent amounts of the C4F6-reactive SOD1. We thus examined effects of the CuSO_4_/ZnSO_4_ addition on the cell viability but found that NSC-34 cells were highly susceptible to the Cu/Zn salts even in the absence of the CSF samples (Additional file 8: Figure S7). For example, when we added 5 μM CuSO_4_ or 5 μM ZnSO_4_ to differentiated NSC-34 cells, the cell viability reduced by 40 and 20%, respectively. Therefore, it is difficult to test the efficacy of CuSO_4_/ZnSO_4_ for alleviation of the toxicity caused by the misfolded SOD1 in CSF.

To test involvement of the misfolded SOD1 in the toxicity, we next examined the viability of differentiated NSC-34 cells by adding the CSF samples from which the misfolded SOD1 was removed by immunoprecipitation with the C4F6-conjugated magnetic beads (Figs. 4 and 5; Additional file 5, Figure S4). We found that the absorption of misfolded SOD1 with C4F6 significantly ameliorated the toxicity of the CSF samples from the ALS cases as well as the PD^SOD1^ and PSP^SOD1^ cases (*P* < 0.01 in ALS w/ C4F6 vs. ALS and also in PD^SOD1^/PSP^SOD1^ w/ C4F6 vs. PD^SOD1^/PSP^SOD1^; Fig. 9a, black filled bars vs. white bars). While residual toxicity (*approx.* 20% reduction in the viability) in the C4F6-treated CSF samples of the ALS, PD^SOD1^, and PSP^SOD1^ cases suggests the presence of unidentified toxic species (*P* < 0.01 in ALS w/ C4F6 vs. non-ND w/ C4F6 and also in PD^SOD1^/PSP^SOD1^ w/ C4F6 vs. non-ND w/ C4F6; *P* > 0.05 in ALS w/ C4F6 vs. PD^SOD1^/PSP^SOD1^ w/ C4F6), the amelioration of the toxicity was not observed with normal mouse IgG instead of C4F6 (*P* > 0.05 in ALS w/ mouse IgG vs. ALS and also in PD^SOD1^/PSP^SOD1^ w/ mouse IgG vs. PD^SOD1^/PSP^SOD1^; the middle and lower panels of Fig. 9a, gray bars vs. white bars). We thus suppose that the C4F6-reactive misfolded SOD1 has significant contributions to the cellular toxicity of CSF of the ALS cases as well as the PD^SOD1^ and PSP^SOD1^ cases.

Microscopic examination of the cells also supports the misfolded SOD1-dependent toxicity of CSF. The cells treated with the CSF samples from the non-ND cases were pleiomorphic with some processes (Fig. 9b), which is a typical morphology of differentiated NSC-34 cells. Also, those cells were not stained with DAPI, which preferentially stains dead cells [39]. Upon addition of the CSF samples from the ALS cases, the cells exhibited smaller, round morphologies and were also stained with DAPI (Fig. 9e). When the CSF samples from the ALS cases were first treated with C4F6 and then added to differentiated NSC-34 cells, large fractions of the cells were not stained with DAPI and were characterized by the morphologies with some processes (Fig. 9g). When treated with mouse IgG, in contrast, the CSF samples from the ALS cases remained toxic to the cells (Fig. 9f). No obvious changes in cellular morphologies were observed with the CSF samples from the non-ND cases after treatment with C4F6 as well as mouse IgG (Fig. 9c and d), confirming little toxicity of CSF from the non-ND cases. Collectively, we suggest that the toxicity of CSF from ALS cases is exerted largely by the misfolded SOD1 proteins that can be absorbed with C4F6.

## Discussion

Misfolded forms of mutant SOD1 proteins in spinal motor neurons have been established as a hallmark of *SOD1*-ALS [58], but pathological involvement of wild-type SOD1 in sALS cases without *SOD1* mutations remained controversial. In this study, we found for the first time that significant amounts of misfolded wild-type SOD1 existed in CSF of all sALS cases examined and also some of the PD and PSP cases. Taking into account reports on abnormal accumulation of misfolded SOD1 in PD brains [16] and also of α-synuclein in ALS spinal cords [59, 60], there would be a shared pathomechanism between ALS and PD; indeed, sporadic and familial cases of motor neuron disease with parkinsonism have been occasionally reported (e.g. [61]).

Despite this, our findings of misfolded SOD1 in CSF raise lots of fresh questions that should be tested in the future; for example, roles of those misfolded SOD1 species in a pathomechanism of ALS and other neurodegenerative diseases remain totally unknown. Misfolded SOD1 in CSF may be a direct cause of the diseases or may form just as a result of the diseases. Certain pathological environment caused by neurodegeneration might trigger misfolding of SOD1 in CSF. In our current study, misfolded SOD1 was detected in CSF of ALS cases but not of non-ND cases in fifties, sixties and seventies (Tables 1 and 2); therefore, it seems unlikely that age is a decisive factor for the formation of misfolded SOD1 in CSF. Nonetheless, effects of aging on amounts of misfolded SOD1 will be examined in more detail in the future. Also, CSF was collected with the lumbar puncture, and the lumbar spinal cord is the most affected in ALS but not in PD/PSP. Although CSF is well known to be fully exchanged/renewed three to four times in a single day [62], high amounts of misfolded SOD1 in CSF of ALS might be somehow related to such proximity between the punctured site and the affected site.

Furthermore, it remains to be tested whether SOD1 becomes misfolded in CSF or the misfolded SOD1 is secreted from certain cells into CSF. CSF of ALS patients has been characterized by significantly increased oxidative stress [63], and such oxidative conditions are known to trigger misfolding of SOD1 proteins in vitro [64]. Alternatively, it is possible that the misfolded SOD1 in CSF is originated from cells in brain and spinal cord. SOD1 was immunocaptured from spinal cord homogenates of sALS patients with C4F6 antibody [13] but not with other misfolded SOD1-specific antibodies, 3H1, 4A1, and A5E5 [17]. While the exact origin of SOD1 in CSF has not been specified yet, various cellular sources can be considered to contribute to the secretion of their SOD1 proteins into interstitial fluid and CSF. Indeed, previous studies have suggested secretion of SOD1 from neuroblastoma SK-N-BE cells by microvesicular granules in ATP-dependent mechanisms [65] and also from NSC-34 cells [66] via exosomes. Mutant but not wild-type SOD1 has been also found to be secreted by neurosecretory vesicles containing chromogranin [24], and recently, extracellular vesicles containing mutant SOD1 were shown to be secreted from astrocytes and neurons, but not microglia, in brain and spinal cord [67]. Misfolded SOD1 might hence be selectively drained from parenchyma of brain and spinal cord into CSF via interstitial fluid and then accumulated in CSF of ALS possibly due to increased secretion of the misfolded SOD1 from cells and/or impaired removal of the misfolded SOD1 from CSF.

Given that CSF communicates with a broad region of the central nervous system, the misfolded SOD1 in CSF might be involved in spreading the disease. Recent studies have shown that the disease as well as the inclusion pathology in mice expressing mutant SOD1 is induced and accelerated by intraspinal injection of SOD1 fibrils [68, 69]. Indeed, SOD1 in vitro formed amyloid-like fibrillar aggregates, which can function as structural templates triggering a seeded growth of fibrillar aggregates of SOD1 [8]. SOD1 fibrils are secreted from an affected neuron and then taken up by contiguous neurons probably through macropinocytosis [12, 70], where a seeding reaction triggers fibrillation of SOD1. Such a cell-to-cell propagation of SOD1 fibrils by the seeding reaction might describe a focal process of ALS; namely, symptoms most often develop in contiguous anatomical regions and thus contralateral side of the body [71]. A substantial proportion of ALS patients is also known to exhibit clinical progression in a non-contiguous pattern, which is represented by symptom development in legs after bulbar onset and vice versa, for example [72, 73]. Furthermore, an important caveat is that SOD1 becomes prone to fibrillization upon reduction of its conserved intramolecular disulfide bond [8]. Disulfide-reduced SOD1 was not detected in CSF; instead, SOD1 in CSF was found to possess the disulfide bond. It thus remains to be tested whether our misfolded SOD1 in CSF of ALS has a propensity to fibrillize. Given high toxicity of the misfolded SOD1, nonetheless, we suspect that CSF-mediated circulation of the misfolded SOD1 throughout the central nervous system contributes to the spread of ALS into non-contiguous regions.

The C4F6-reactive misfolded SOD1 exerted its toxic effects on cells through unknown mechanisms, but the toxicity was observed at quite low levels of the misfolded SOD1. As shown in Fig. 5, the band intensity of the immunoprecipitated SOD1 (“Bound” fraction) was compared with that of recombinant SOD1 in a fixed amount; thereby, the CSF samples of the ALS cases (40 μL) were found to contain approximately 0.2 ng of the C4F6-reactive SOD1 as rough estimation, corresponding to the molar concentration of approximately 0.3 nM. In our viability assay using NSC-34 cells, 10% (v/v) CSF was added to culture media; therefore, C4F6-reactive SOD1 could exert its toxicity in a picomolar range. While it needs to be clarified whether levels of the misfolded SOD1 in CSF correlate with disease progression, removal of the misfolded SOD1 from CSF would reduce its toxicity. In that sense, it is notable that, through the arachnoid villi, CSF is ultimately drained to vasculature, where IgM antibodies recognizing aberrantly oxidized SOD1 have been detected in a subset of sALS patients [74]. The patients with anti-oxidized SOD1 IgM antibodies survived longer (6.4 years) than patients lacking these antibodies [74]. Recently, antibodies selectively recognizing misfolded SOD1 were also generated by screening human memory B cells from healthy elderly subjects and were reported to ameliorate motor symptoms of mouse models of ALS expressing mutant SOD1 [75]. Furthermore, we have noted previous attempts of CSF filtration (or called liquorpheresis) to ALS patients albeit with subjective but no objective improvement of disease symptoms [76, 77]. In some of patients with Guillain-Barre syndrome and multiple sclerosis, which are autoimmune diseases affecting the central nervous system or nerve roots, the liquorpheresis improved disease symptoms to some extent without adverse events or complications [78, 79]. For the treatment of ALS, the filtration alone would not remove the misfolded SOD1 from CSF, and a device absorbing the misfolded SOD1 can hence be implemented in the CSF filtration system [78]. The SOD1 misfolding in CSF of sALS and the other neurodegenerative diseases has not been well characterized and is just emerging; therefore, more data need to be accumulated for development of the CSF-based cures of ALS.

## Conclusions

In summary, we found for the first time that most of wild-type SOD1 proteins assume the misfolded conformation(s) in CSF of the ALS cases regardless of *SOD1* mutations. The misfolding of SOD1 in CSF is considered to expose the protein interior covered by Loops IV and VII. Importantly, furthermore, we showed that the toxicity of CSF from the ALS cases was significantly alleviated by removal of the misfolded SOD1 with immunoprecipitation. We hence propose that the misfolded SOD1 in CSF is a pathological species commonly observed in ALS cases both with and without *SOD1* mutations. Removal of the misfolded SOD1 from CSF might be an effective method to alleviate the disease symptoms.

## Supplementary information

**Additional file 1: Table S1.** Primers for PCR amplification/sequencing of *SOD1* exons and a GGGGCC repeat in *C9ORF72.*

**Additional file 2: Figure S1.** Concentrations of total proteins in CSF. The total protein concentrations in CSF were measured with Micro BCA Protein Assay Kit (Thermo Scientific) and compared between ALS (*n* = 21) and non-ALS cases (*n* = 40). The averages are shown as bars. The Student’s *t*-test suggested no significant differences in total protein concentrations of the CSF samples between non-ALS and ALS cases (*P* = 0.10).

**Additional file 3: Figure S2.** Standard curves for sandwich ELISA using misfolded SOD1-specific antibodies. Indicated amounts of recombinant SOD1 proteins (100 μL of a reaction solution) were analyzed by sandwich ELISA using a capture antibody (A, C4F6; B, UβB; C, EDI; D, apoSOD; E, 24–39; F, FL-154). While detailed experimental methods for sandwich ELISA were described in the Methods, positive standards for ELISA were used as follows; G37R-mutant apo-SOD1^S-S^ for C4F6, A4V-mutant apo-SOD1^S-S^ for UβB/EDI, G85R-mutant apo-SOD1^S-S^ for apoSOD, wild-type apo-SOD1^S-S^ for 24–39, and wild-type holo-SOD1^S-S^ for FL-154. Absorbance values at 490 nm in sandwich ELISA were plotted against amounts of SOD1 applied on a well, which is represented as a logarithmic scale. Data (0.01 ng – 10 ng of SOD1 proteins) were fitted to an exponential function, which was shown with an R-squared in each panel. Absorbance values at 490 nm for the preparation of the standard curves (0.01 ng – 100 ng of recombinant SOD1 proteins) were available in Additional file 9.

**Additional file 4: Figure S3.** Selectivity of C4F6 antibody toward mutant/misfolded SOD1. Sandwich ELISA was performed as described in the text (see Methods). Briefly, C4F6 (1:500 dilution) antibody was first adsorbed on wells of an ELISA plate, and recombinant apo-SOD1 variants (WT, A4V, G37R, G85R, and G93A) with the disulfide bond (0.1 μg) was then applied to the well. Pan-SOD1 (1:500 dilution) and anti-sheep secondary antibody (1:500 dilution) were used as detection and secondary antibodies, respectively. Three independent experiments were performed to estimate error bars (standard deviation).

**Additional file 5: Figure S4.** Almost complete depletion of C4F6-reactive SOD1 from CSF by immunoprecipitation. C4F6-crosslinked magnetic beads were first incubated with the CSF of ALS2 (40 μL) at 4 °C for 24 h, and the solution was collected as the “unbound” fraction in 1st IP. The remaining magnetic beads were then treated with 10 μL of 100 mM citrate buffer at pH 3.1, and the eluate was collected as the “bound” fraction in 1st IP. The unbound fraction (40 μL) in the 1st IP step was further treated with C4F6-crosslinked magnetic beads at 4 °C for 24 h, and the solution was collected as the unbound fraction in 2nd IP. The remaining magnetic beads were then treated with 10 μL of 100 mM citrate buffer at pH 3.1, and the eluate was collected as the bound fraction in 2nd IP. Ten microliter of the CSF (input) as well as the unbound and bound fractions in 1st IP and 2nd IP steps were analyzed by Western blotting with FL-154 antibody. The full-length and truncated SOD1 proteins were indicated with filled and open arrows, respectively.

**Additional file 6: Figure S5.** Reactivities of SOD1 antibodies toward conformationally distinct states of SOD1 in vitro were examined by indirect ELISA. Recombinant SOD1 proteins (black, wild-type: white, A4V: red, G37R: blue, G85R) were prepared in the following states: +Cu/Zn S-S, SOD1 with the disulfide bond (SOD1^S-S^) in the presence of copper and zinc ions; Apo S-S, SOD1^S-S^ in the absence of any metal ions; Apo SH, SOD1 without the disulfide bond (SOD1^SH^) in the absence of any metal ions; SS oligomer, SOD1 oligomers crosslinked via disulfide bonds prepared from Apo S-S; Aggregates, insoluble SOD1 aggregates prepared from Apo SH. The experimental methods to prepare those SOD1 proteins can be found in our previous papers (ref #7, 8). Proteins (5 μg) were first adsorbed in wells of an ELISA plate and then detected with 0.2 μg/mL of (A) UβB, (B) EDI, (C) SOD1^int^, (D) apoSOD, (E) 24–39, and (F) Pan-SOD1 antibodies. A detailed procedure for indirect ELISA can be found in our previous papers (ref #35, 36), which have also reported the data on UβB, EDI, SOD1^int^, and apoSOD. Three independent experiments were performed to estimate error bars (standard deviation).

**Additional file 7: Figure S6.** Addition of metal salts has little effects on amounts of SOD1 adsorbed on wells of an ELISA plate. The experiments were performed as described in Fig. 8B except the capture antibody. Briefly, the CSF samples (20 μg of total proteins, 100 μL of total volume) were first incubated with either (blue bars) 100 μM CuSO_4_ or (red bars) 100 μM ZnSO_4_ at 4 °C overnight and then analyzed by sandwich ELISA with FL-154 and Pan-SOD1 as capture and detection antibodies, respectively. As a control, the CSF samples incubated at 4 °C overnight without addition of any metal ions (black bars) were also examined. The experiments were performed in triplicate to estimate error bars (standard deviation).

**Additional file 8: Figure S7.** Effects of metal salts addition on the viability of differentiated NSC-34 cells. Differentiated NSC-34 cells were first prepared by the method described in the text, and either CuSO_4_ or ZnSO_4_ in the indicated concentration was then added. After incubation for 48 h, the viability of the NSC-34 cells was assayed with Cell Counting Kit-8 (Dojindo) and represented as the relative viability to the one in the absence of the metal salt addition. The data were represented as the averaged cell viability relative to that of the negative control in which neither CuSO_4_ nor ZnSO_4_ was added. Three independent experiments were performed to estimate error bars (standard deviation).

**Additional file 9.** A spreadsheet with ELISA data was available in a Microsoft Excel file. In a “Standard curve” sheet, absorbance values at 490 nm for preparation of standard curves (i.e. Additional file 3: Figure S2) were summarized. Also, absorbance values at 490 nm in the sandwich ELISA on CSF were available in a “Absorbance” sheet. As described in the Methods, furthermore, absorbance values at 490 nm measured in the ELISA were substituted into equations fitted to standard curves (Additional file 3: Figure S2), and apparent amounts of SOD1 were summarized in an “Apparent amount” sheet (i.e. Figures 3 and 6). In all sheets, averages together with standard deviations of the observed data were shown.

## Data Availability

The datasets during and/or analyzed during the current study available from the corresponding author on reasonable request.

## Abbreviations

AD: Alzheimer’s disease
ALS: Amyotrophic lateral sclerosis
CSF: Cerebrospinal fluid
DLB: Dementia with Lewy bodies
Non-ND: Non-neurodegenerative disease
PD: Parkinson’s disease
PD^SOD1^: PD with misfolded SOD1
PSP: Progressive supranuclear palsy
PSP^SOD1^: PSP with misfolded SOD1
sALS: Sporadic ALS
SOD1: Cu/Zn-superoxide dismutase
*SOD1*-ALS: ALS with mutations in the *SOD1* gene
SOD1^SO3H^: SOD1 sulfonylated at Cys111
