# Supplementary material for: Wild-type Cu/Zn-superoxide dismutase is misfolded in cerebrospinal fluid of sporadic amyotrophic lateral sclerosis
Source: Mol Neurodegener. 2019 Nov 19;14:42. doi: 10.1186/s13024-019-0341-5 (PMC6862823; doi:10.1186/s13024-019-0341-5)
Supplement: Supplementary file 1 — Additional file 1: Table S1. Primers for PCR amplification/sequencing of SOD1 exons and a GGGGCC repeat in C9ORF72. [file 13024_2019_341_MOESM1_ESM.pdf]

Table S1 Primers for PCR amplification/sequencing of *SOD1* exons and a GGGGCC repeat in *C9ORF72*

	Primer	Sequence (5’ -> 3’)	Primer for sequencing (5’ -> 3’)
Exon 1	Ex1_fwd	CGGAGGTCTGGCCTATAAAGTAGTCGCGGAGAC	Ex1_rev2
	Ex1_rev	GATCTTCAAATAAGCTAACTGTGCACCCAAAG	CTGACCTAGAGCGCTGAAGCCGGAAAGCGG
Exon 2	Ex2_fwd	ATTTGGACACAGATTTTTCCACTCCCAAGTCTGGC	Ex2_rev2
	Ex2_rev	GTTTTCCCAGGCACAGTGGCTCACACATGTAATC	GGGTTTTAACGTTTAGGGGC
Exon 3	Ex3_fwd	CAGAAGTCGTGATGCAGGTCAGCACTTTCTCCATG	Ex3_fwd
	Ex3/4_rev	GGGACAACAGCTTCCCACAAGTTAAACAAGTC	CAGAAGTCGTGATGCAGGTCAGCACTTTCTCCATG
Exon 4	Ex3_fwd	CAGAAGTCGTGATGCAGGTCAGCACTTTCTCCATG	Ex4_fwd
	Ex3/4_rev	GGGACAACAGCTTCCCACAAGTTAAACAAGTC	GAAGCCTTGTTTGAAGAGCTGTATTTAGAATGCC
Exon 5	Ex4_fwd	GAAGCCTTGTTTGAAGAGCTGTATTTAGAATGCC	Ex4/5_rev
	Ex4/5_rev	CAGGTACTTTAAAGCAACTCTGAAAAAGTCACAC	CAGGTACTTTAAAGCAACTCTGAAAAAGTCACAC
C9ORF72	repeat_fwd	CCTGTAGCAAGCTCTGGAACTCAGG	---
	repeat_rev	AGTAAAAATGCGTCGAGCTCTGAGG	---
