# Supplementary figures and images for: Wild-type Cu/Zn-superoxide dismutase is misfolded in cerebrospinal fluid of sporadic amyotrophic lateral sclerosis
Source: Mol Neurodegener. 2019 Nov 19;14:42. doi: 10.1186/s13024-019-0341-5 (PMC6862823; doi:10.1186/s13024-019-0341-5)

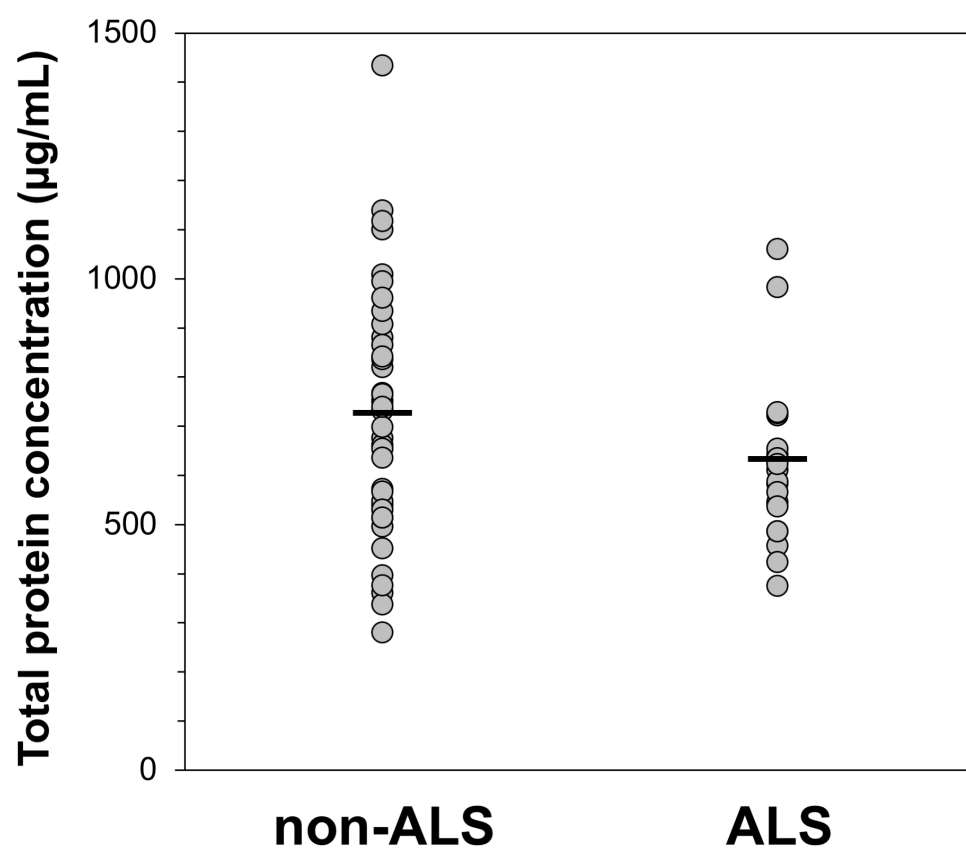

Supplement: Supplementary file 2 — Additional file 2: Figure S1. Concentrations of total proteins in CSF. The total protein concentrations in CSF were measured with Micro BCA Protein Assay Kit (Thermo Scientific) and compared between ALS (n = 21) and non-ALS cases (n = 40). The averages are shown as bars. The Student’s t-test suggested no significant differences in total protein concentrations of the CSF samples between non-ALS and ALS cases (P = 0.10). [file 13024_2019_341_MOESM2_ESM.pdf]

**(A) C4F6**

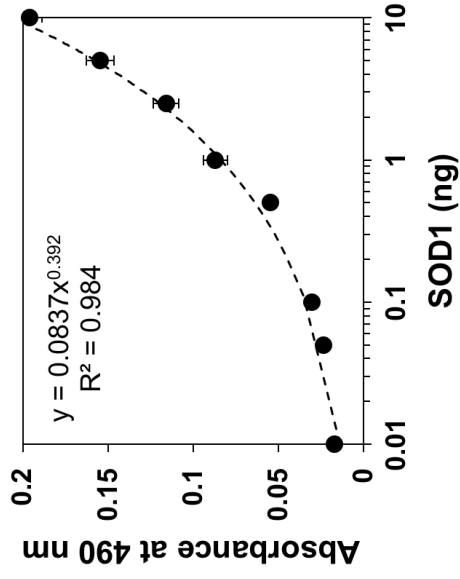

**(B) U $\beta$ B**

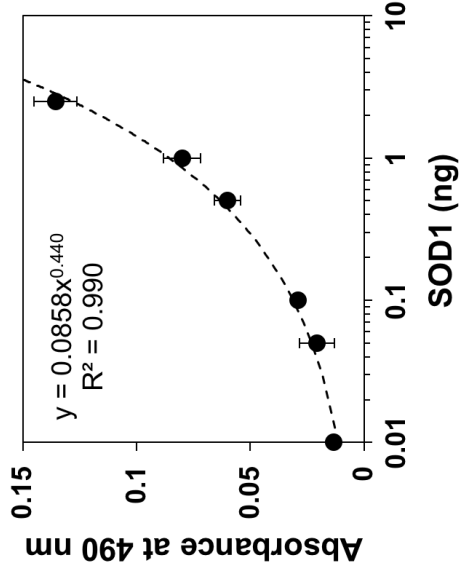

**(C) EDI**

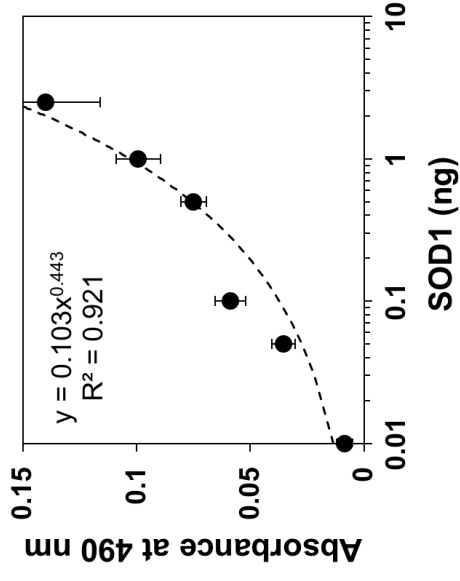

**(D) apoSOD**

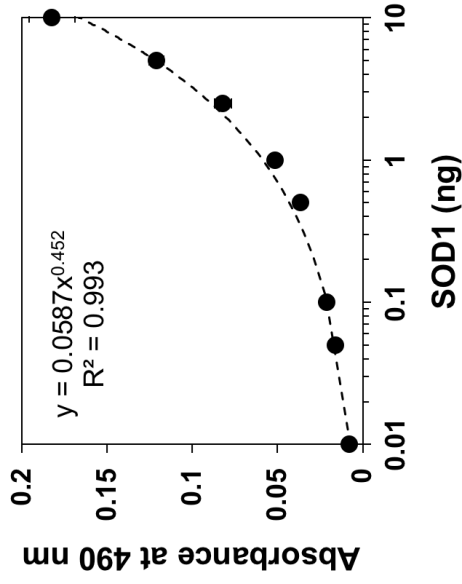

**(E) 24-39**

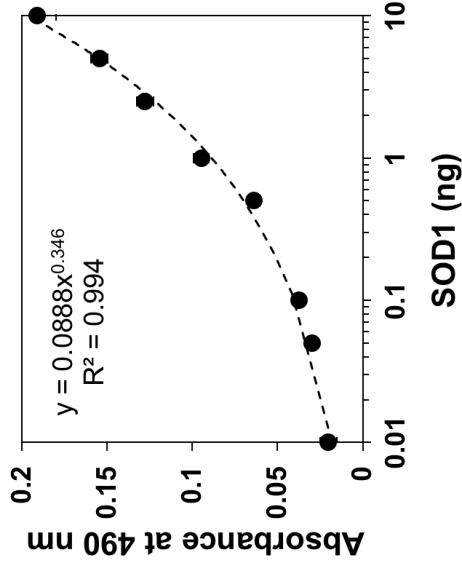

**(F) FL-154**

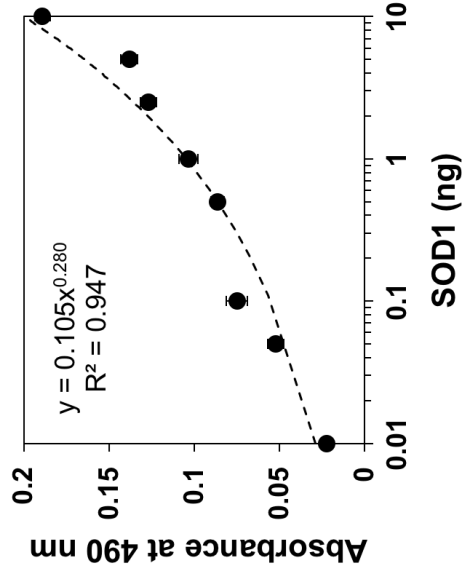

Supplement: Supplementary file 3 — Additional file 3: Figure S2. Standard curves for sandwich ELISA using misfolded SOD1-specific antibodies. Indicated amounts of recombinant SOD1 proteins (100 μL of a reaction solution) were analyzed by sandwich ELISA using a capture antibody (A, C4F6; B, UβB; C, EDI; D, apoSOD; E, 24–39; F, FL-154). While detailed experimental methods for sandwich ELISA were described in the Methods, positive standards for ELISA were used as follows; G37R-mutant apo-SOD1S-S for C4F6, A4V-mutant apo-SOD1S-S for UβB/EDI, G85R-mutant apo-SOD1S-S for apoSOD, wild-type apo-SOD1S-S for 24–39, and wild-type holo-SOD1S-S for FL-154. Absorbance values at 490 nm in sandwich ELISA were plotted against amounts of SOD1 applied on a well, which is represented as a logarithmic scale. Data (0.01 ng – 10 ng of SOD1 proteins) were fitted to an exponential function, which was shown with an R-squared in each panel. Absorbance values at 490 nm for the preparation of the standard curves (0.01 ng – 100 ng of recombinant SOD1 proteins) were available in Additional file 9. [file 13024_2019_341_MOESM3_ESM.pdf]

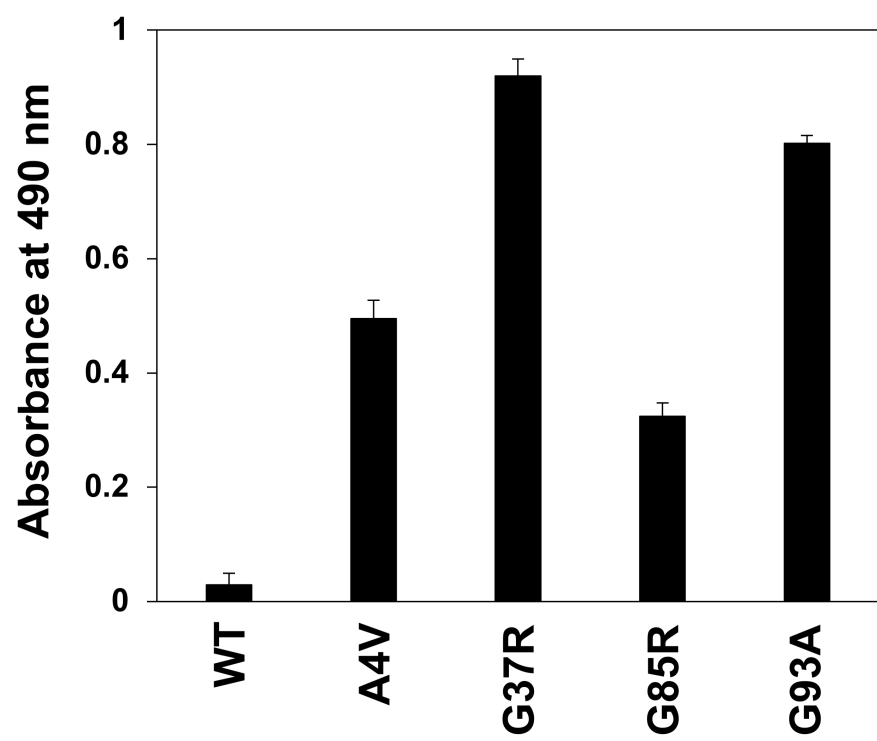

Supplement: Supplementary file 4 — Additional file 4: Figure S3. Selectivity of C4F6 antibody toward mutant/misfolded SOD1. Sandwich ELISA was performed as described in the text (see Methods). Briefly, C4F6 (1:500 dilution) antibody was first adsorbed on wells of an ELISA plate, and recombinant apo-SOD1 variants (WT, A4V, G37R, G85R, and G93A) with the disulfide bond (0.1 μg) was then applied to the well. Pan-SOD1 (1:500 dilution) and anti-sheep secondary antibody (1:500 dilution) were used as detection and secondary antibodies, respectively. Three independent experiments were performed to estimate error bars (standard deviation). [file 13024_2019_341_MOESM4_ESM.pdf]

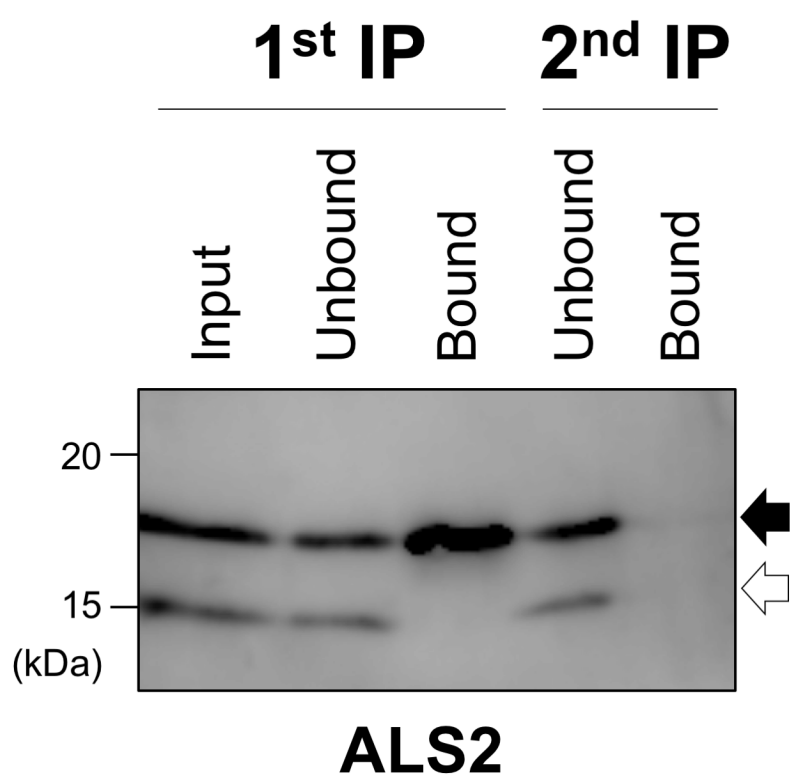

Supplement: Supplementary file 5 — Additional file 5: Figure S4. Almost complete depletion of C4F6-reactive SOD1 from CSF by immunoprecipitation. C4F6-crosslinked magnetic beads were first incubated with the CSF of ALS2 (40 μL) at 4 °C for 24 h, and the solution was collected as the “unbound” fraction in 1st IP. The remaining magnetic beads were then treated with 10 μL of 100 mM citrate buffer at pH 3.1, and the eluate was collected as the “bound” fraction in 1st IP. The unbound fraction (40 μL) in the 1st IP step was further treated with C4F6-crosslinked magnetic beads at 4 °C for 24 h, and the solution was collected as the unbound fraction in 2nd IP. The remaining magnetic beads were then treated with 10 μL of 100 mM citrate buffer at pH 3.1, and the eluate was collected as the bound fraction in 2nd IP. Ten microliter of the CSF (input) as well as the unbound and bound fractions in 1st IP and 2nd IP steps were analyzed by Western blotting with FL-154 antibody. The full-length and truncated SOD1 proteins were indicated with filled and open arrows, respectively. [file 13024_2019_341_MOESM5_ESM.pdf]

(A) U $\beta$ B

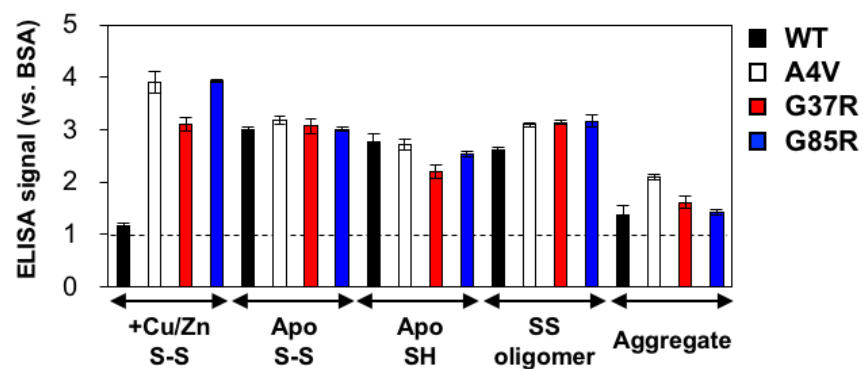

(D) apoSOD

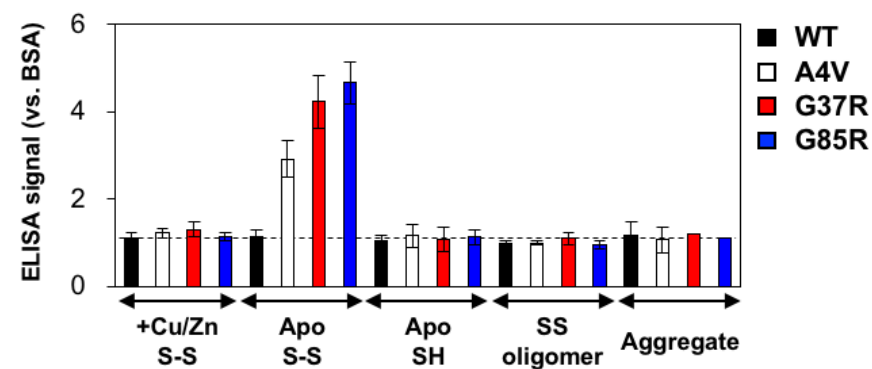

(B) EDI

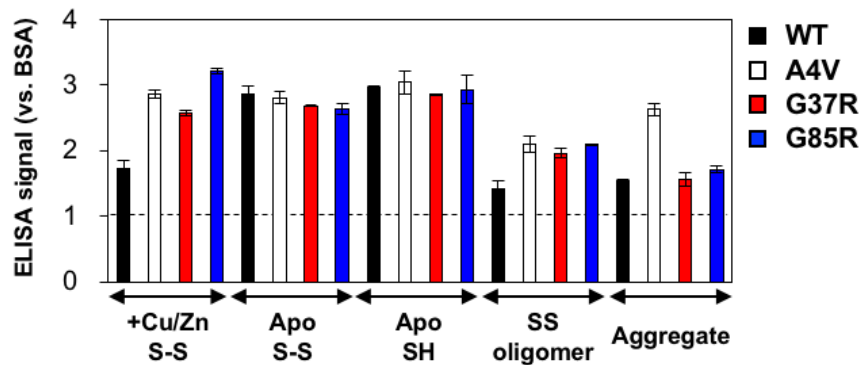

(E) 24-39

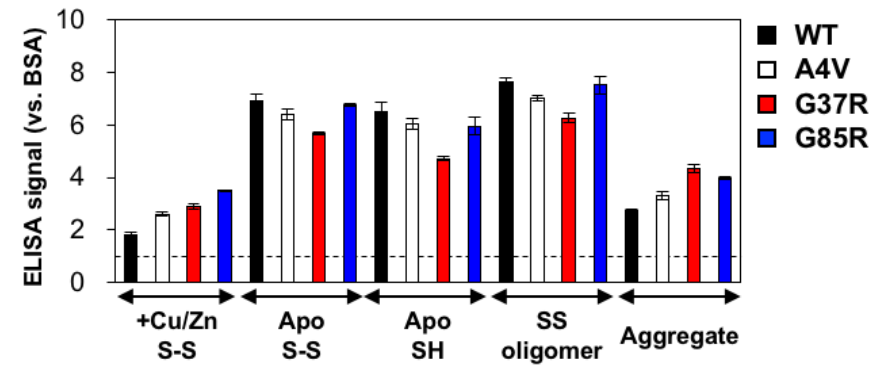

(C) SOD1<sup>int</sup>

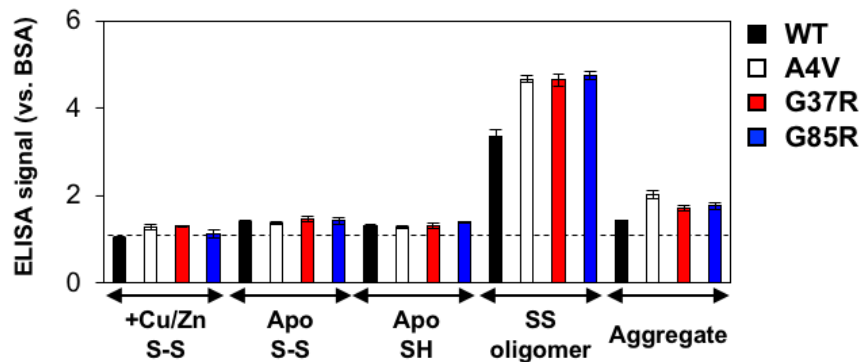

(F) Pan-SOD1

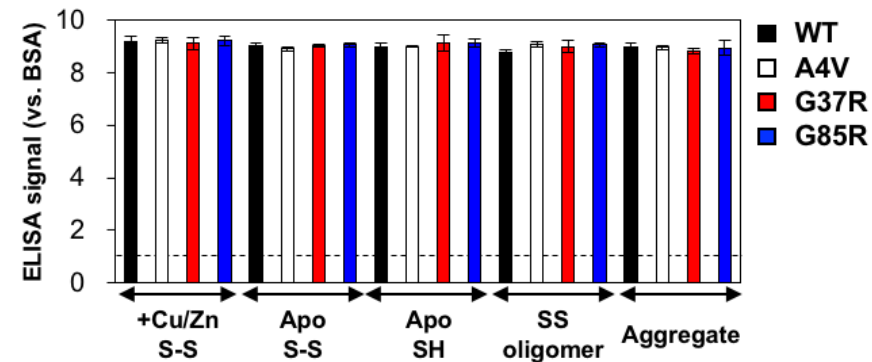

Supplement: Supplementary file 6 — Additional file 6: Figure S5. Reactivities of SOD1 antibodies toward conformationally distinct states of SOD1 in vitro were examined by indirect ELISA. Recombinant SOD1 proteins (black, wild-type: white, A4V: red, G37R: blue, G85R) were prepared in the following states: +Cu/Zn S-S, SOD1 with the disulfide bond (SOD1S-S) in the presence of copper and zinc ions; Apo S-S, SOD1S-S in the absence of any metal ions; Apo SH, SOD1 without the disulfide bond (SOD1SH) in the absence of any metal ions; SS oligomer, SOD1 oligomers crosslinked via disulfide bonds prepared from Apo S-S; Aggregates, insoluble SOD1 aggregates prepared from Apo SH. The experimental methods to prepare those SOD1 proteins can be found in our previous papers (ref #7, 8). Proteins (5 μg) were first adsorbed in wells of an ELISA plate and then detected with 0.2 μg/mL of (A) UβB, (B) EDI, (C) SOD1int, (D) apoSOD, (E) 24–39, and (F) Pan-SOD1 antibodies. A detailed procedure for indirect ELISA can be found in our previous papers (ref #35, 36), which have also reported the data on UβB, EDI, SOD1int, and apoSOD. Three independent experiments were performed to estimate error bars (standard deviation). [file 13024_2019_341_MOESM6_ESM.pdf]

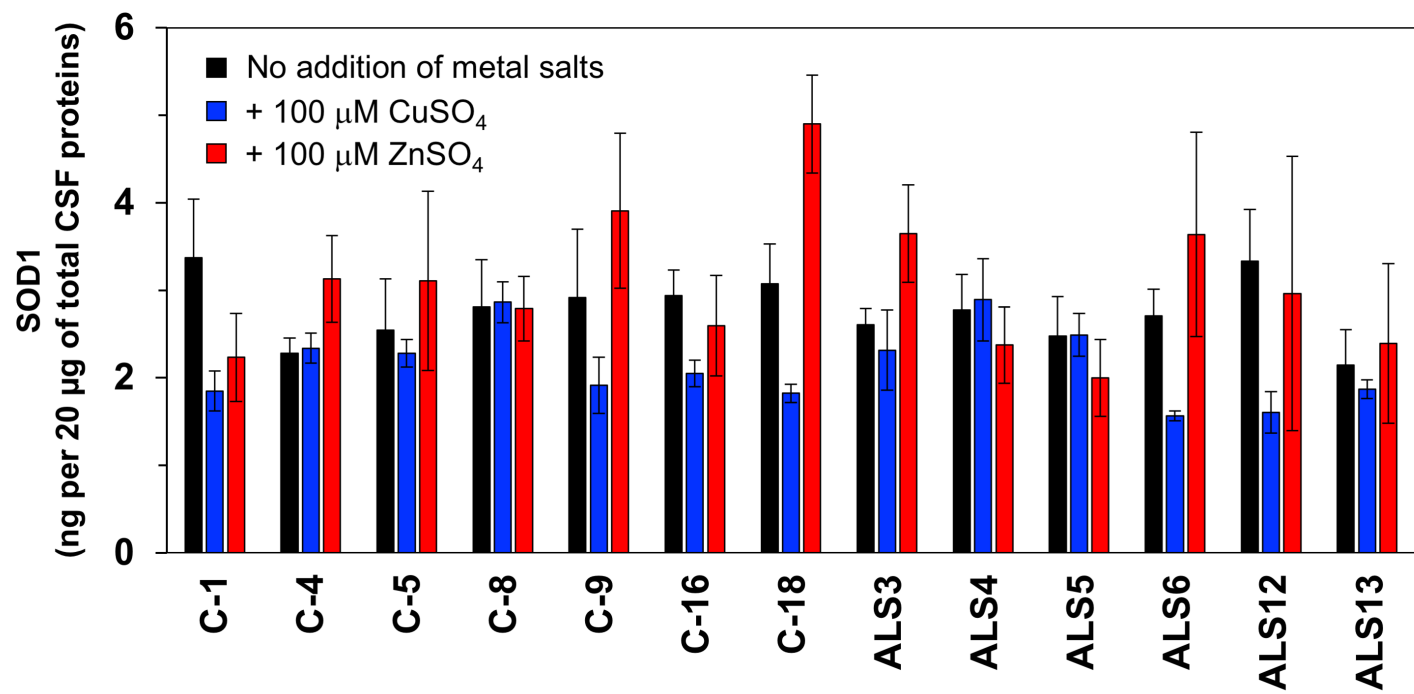

Supplement: Supplementary file 7 — Additional file 7: Figure S6. Addition of metal salts has little effects on amounts of SOD1 adsorbed on wells of an ELISA plate. The experiments were performed as described in Fig. 8B except the capture antibody. Briefly, the CSF samples (20 μg of total proteins, 100 μL of total volume) were first incubated with either (blue bars) 100 μM CuSO4 or (red bars) 100 μM ZnSO4 at 4 °C overnight and then analyzed by sandwich ELISA with FL-154 and Pan-SOD1 as capture and detection antibodies, respectively. As a control, the CSF samples incubated at 4 °C overnight without addition of any metal ions (black bars) were also examined. The experiments were performed in triplicate to estimate error bars (standard deviation). [file 13024_2019_341_MOESM7_ESM.pdf]

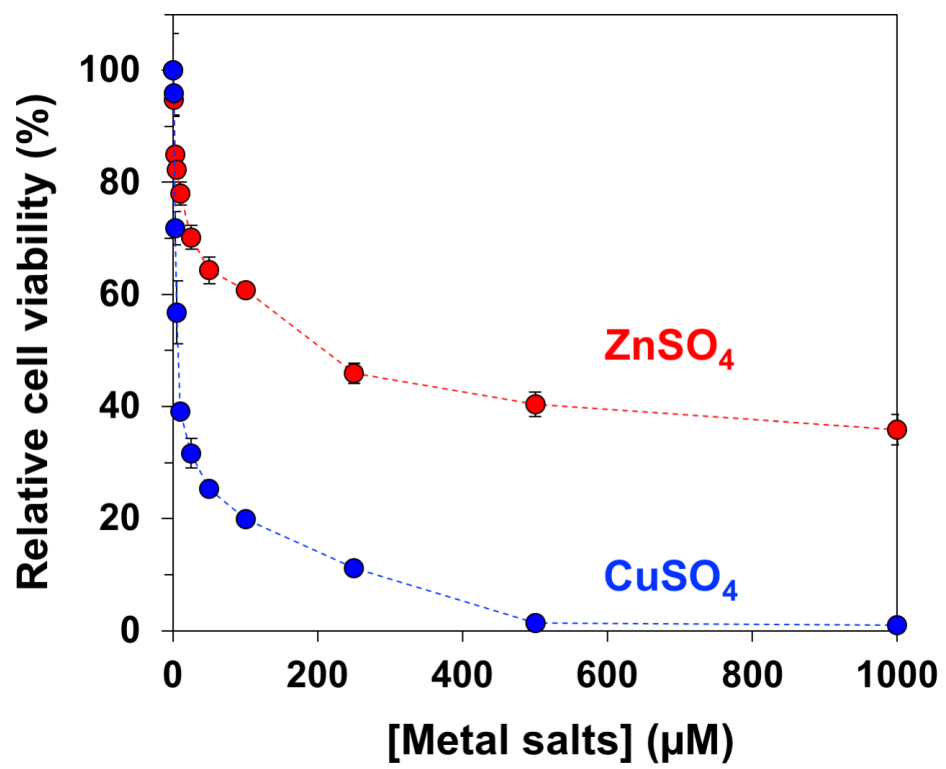

Supplement: Supplementary file 8 — Additional file 8: Figure S7. Effects of metal salts addition on the viability of differentiated NSC-34 cells. Differentiated NSC-34 cells were first prepared by the method described in the text, and either CuSO4 or ZnSO4 in the indicated concentration was then added. After incubation for 48 h, the viability of the NSC-34 cells was assayed with Cell Counting Kit-8 (Dojindo) and represented as the relative viability to the one in the absence of the metal salt addition. The data were represented as the averaged cell viability relative to that of the negative control in which neither CuSO4 nor ZnSO4 was added. Three independent experiments were performed to estimate error bars (standard deviation). [file 13024_2019_341_MOESM8_ESM.pdf]
